# Neural correlates of high-risk behavior tendencies and impulsivity in an emotional Go/NoGo fMRI task

**DOI:** 10.3389/fnsys.2015.00024

**Published:** 2015-03-10

**Authors:** Matthew R. G. Brown, James R. A. Benoit, Michal Juhás, R. M. Lebel, Marnie MacKay, Ericson Dametto, Peter H. Silverstone, Florin Dolcos, Serdar M. Dursun, Andrew J. Greenshaw

**Affiliations:** ^1^Department of Psychiatry, University of AlbertaEdmonton, AB, Canada; ^2^Department of Biomedical Engineering, University of AlbertaEdmonton, AB, Canada; ^3^Department of Psychology, Neuroscience Program, and the Beckman Institute for Advanced Science and Technology, University of Illinois Urbana-ChampaignUrbana-Champaign, IL, USA

**Keywords:** high-risk behavior, impulsivity, emotional Go/NoGo, CARE, BIS, fMRI

## Abstract

Improved neuroscientific understanding of high-risk behaviors such as alcohol binging, drug use, and unsafe sex will lead to therapeutic advances for high-risk groups. High-risk behavior often occurs in an emotionally-charged context, and behavioral inhibition and emotion regulation play important roles in risk-related decision making. High impulsivity is an important potential contributor to high-risk behavior tendencies. We explored the relationships between high-risk behavior tendencies, impulsivity, and fMRI brain activations in an emotional Go/NoGo task. This task presented emotional distractor pictures (aversive vs. neutral) simultaneously with Go/NoGo stimuli (square vs. circle) that required a button press or withholding of the press, respectively. Participants' risk behavior tendencies were assessed with the Cognitive Appraisal of Risky Events (CARE) scale. The Barratt Impulsivity Scale 11 (BIS) was used to assess participant impulsivity. Individuals with higher CARE risk scores exhibited reduced activation related to response inhibition (NoGo−Go) in right orbital frontal cortex (OFC) and ventromedial prefrontal cortex. These regions did not show a significant relationship with impulsivity scores. Conversely, more impulsive individuals showed reduced emotion-related activity (aversive−neutral distractors) in dorsomedial prefrontal cortex, perigenual anterior cingulate cortex, and right posterior OFC. There were distinct neural correlates of high-risk behavior tendency and impulsivity in terms of brain activity in the emotional Go/NoGo task. This dissociation supports the conception of high-risk behavior tendency as a distinct construct from that of impulsivity. Our results suggest that treatment for high-risk behavior may be more effective with a nuanced approach that does not conflate high impulsivity necessarily with high-risk behavior tendencies.

## 1. Introduction

High-risk behaviors such as binge drinking, substance abuse, unsafe sex, and physical violence create increased potential for harm to mental and physical health and general well-being (Jessor, 1991; Arnett, 1992; Blakemore and Robbins, 2012). High-risk behaviors account for a substantial proportion of deaths and injuries among adolescents and young adults (NCHS, 2007; Statistics Canada, 2010; Viner et al., 2012) as well as poor health outcomes in later life (Anda et al., 2006; Eaton et al., 2006; Hawton and O'Connor, 2012). In addition to personal costs, high-risk behaviors impose large economic costs on society (MacKersie et al., 1995), for example in health services. Improved understanding of the neurobiology of high-risk behavior is important and shows promise for improved treatments and policies for addressing high-risk behavior.

Many high-risk behaviors occur in emotionally-charged circumstances such as “wild” parties or interpersonal confrontations. High-risk behavior is complex, but behavioral inhibition, emotional responses, and emotion regulation are thought to play important roles in risk-related decision making in many such circumstances. Previous studies have emphasized individual differences and developmental changes in impulsivity and emotion processing as important factors contributing to high-risk behavior tendencies (Jessor, 1991; Arnett, 1992, 1994, 1996; Ernst et al., 2006; Steinberg, 2007; Casey et al., 2008; Ernst and Mueller, 2008; Gullo and Dawe, 2008; Steinberg, 2008; Ernst and Fudge, 2009; Romer et al., 2009; Romer, 2010; Casey et al., 2011; Dalley et al., 2011; Mitchell, 2011; Blakemore and Robbins, 2012; Whelan et al., 2012; Bari and Robbins, 2013). In addition to circumstantial decision making, there is an important interplay between high-risk behaviors, emotional dysregulation, and impulsive decision making in a clinical context, for example in borderline personality disorder (BPD), attention-deficit/hyperactivity disorder (ADHD), obsessive-compulsive disorder (OCD), substance use disorder (SUD), pathological gambling, and bulimia nervosa (Aron and Poldrack, 2006; Chamberlain and Sahakian, 2007; Kemps and Wilsdon, 2010; Reid et al., 2014; Sebastian et al., 2014). In this study, we investigate impulsivity as it relates to high-risk behavior and fMRI brain activity patterns associated with behavioral control in emotional contexts.

Impulsivity is a complex construct, and there are multiple proposals on the ontology of impulsivity and its different possible components and sub-processes (see Whiteside and Lynam, 2001; Dalley et al., 2011; Bari and Robbins, 2013). Dalley et al. (2011) define impulsivity informally as “the tendency to act prematurely without foresight.” Impulsivity can be operationally measured using self-report instruments such as the Barratt Impulsivity Scale (BIS; Barratt, 1959; Patton et al., 1995). Other widely-used instruments that assess impulsivity, subcomponents of impulsivity, or constructs related to impulsivity include the Urgency, Premeditation, Perseverance, and Sensation Seeking Scale (UPPS; Whiteside and Lynam, 2001); the Sensation Seeking Scale (SSS; Zuckerman, 1994); the Tridimensional Personality Questionnaire (TPQ; Cloninger et al., 1991); and the I-7 Impulsiveness Questionnaire (I7; Eysenck et al., 1985). The choice of questionnaire used to assess impulsivity implies a certain conception of the impulsivity construct. For example, the UPPS includes sensation seeking as a subcomponent of impulsivity, whereas the BIS does not include sensation seeking as a subscale nor does it include the sort of questions used to assess sensation seeking in the UPPS or SSS. At present, there is no consensus on a single, “correct” version of the impulsivity construct. We focus on impulsivity as captured by the BIS instrument, while acknowledging that other conceptions also provide valuable perspective and insight.

The BIS and other impulsivity scales provide numerical scores for an individual's overall impulsivity level, as well as subscores for various subcomponents of impulsivity. Greater impulsivity scores on standardized self-report questionnaires are known to be associated with increased risk behavior tendencies (Levitt, 1991; Moore and Rosenthal, 1993; Luengo et al., 1994; Stanford et al., 1996; Cyders et al., 2007; Gullo and Dawe, 2008; Romer et al., 2009; Zapolski et al., 2009; Romer, 2010; Dalley et al., 2011; Mishra and Lalumière, 2011; Christiansen et al., 2012; Stautz and Cooper, 2013). However, it is noteworthy that at least one dissociation between risk behavior tendencies, in this case smoking tendencies, and impulsivity has been reported (Ryan et al., 2013). In addition to impulsivity, other contributors to high-risk behavior have been proposed, such as reward seeking and sensation seeking (see Romer et al., 2009; Romer, 2010; Dalley et al., 2011; Blakemore and Robbins, 2012).

Individual differences in impulsivity may be related to differences in cognitive control of behavior, emotions, and other mental processes (Dalley et al., 2011; Bari and Robbins, 2013). Inhibition has been suggested to be an important component of cognitive control (see Ainslie, 1975; Smith, 1992; Dempster and Brainerd, 1995; Aron, 2007), with response inhibition being one widely-studied example of inhibition (see Wager et al., 2005; Aron, 2007). The classic Go/NoGo task (Donders, 1868/1969) provides a means of recruiting and investigating response inhibition processes. This task presents the participant with frequent Go stimuli requiring a button press as well as rare NoGo stimuli requiring inhibition of the button press. The button press response is made automatic, or prepotent, by the frequent Go trials, requiring the participant to actively inhibit that response in NoGo trials. Psychometric measures of impulsivity, such as the BIS, do not correlate significantly with behavioral performance measures on the Go/NoGo task, including reaction times and error rates (Horn et al., 2003; Asahi et al., 2004; Reynolds et al., 2006, 2008; Christiansen et al., 2012). However, Dalley et al. (2011) differentiate between impulsivity based on motor disinhibition from that based on temporal discounting. It is possible that psychometrically-derived behavioral impulsivity may be more related to temporal discounting, while poor Go/NoGo performance may be more related to motor disinhibition. Nonetheless, psychometric impulsivity measures have been shown to be correlated with changes in fMRI activation patterns evoked by the Go/NoGo task. Individuals with greater Barratt Impulsivity Scores were found to exhibit less response inhibition-related fMRI activation in right dorsolateral prefrontal cortex (dlPFC) (Asahi et al., 2004) and in dorsomedial prefrontal cortex (dmPFC) (Horn et al., 2003). Horn et al. (2003) also found that scores on Eysenck's Impulsivity Scale were positively correlated with response inhibition-related activation in right ventrolateral prefrontal cortex (vlPFC^1^). Therefore, there is evidence in the literature that individual differences in psychometrically-measured impulsivity may be related to differences in recruitment of cognitive processes during Go/NoGo task performance.

This study examined the relationships between participants' high-risk behavior tendencies, levels of impulsivity, and recruitment of response inhibition and emotional stimulus processing during performance on an emotional Go/NoGo task using functional magnetic resonance imaging (fMRI). The emotional Go/NoGo task used here presented a distractor image that was emotionally neutral or aversive simultaneously with each Go or NoGo stimulus. This task also allowed us to investigate response inhibition specifically in aversive emotional contexts by comparing NoGo vs. Go activation in the presence of aversive distractor images.

A variety of prefrontal brain regions are thought to have roles in the executive and emotion processing needed to perform this emotional Go/NoGo task. dlPFC, vlPFC, orbitofrontal cortex (OFC), and ventromedial PFC (vmPFC) are involved in response inhibition in the Go/NoGo task as well as inhibition in other executive control tasks (see Aron et al., 2004a, 2007; Dolcos et al., 2011; Mitchell, 2011; Mahmood et al., 2013). Anterior cingulate cortex (ACC) has been implicated in error detection and conflict monitoring in the Go/NoGo and other cognitive tasks (Carter et al., 1998, 1999; Garavan et al., 1999; Botvinick et al., 2004; Kerns et al., 2004; Brown and Braver, 2005; Mitchell, 2011). Dorsomedial PFC (dmPFC) may also contribute to response conflict processing (see Ridderinkhof et al., 2004) as well as to response selection and response inhibition in the Go/NoGo task (Simmonds et al., 2008). dmPFC is also thought be involved in resolution of response conflict and outcome value-related aspects of decision making (Venkatraman et al., 2009). OFC and vlPFC are thought to be involved in processing emotional stimuli, for example to evaluate valence (Dolcos et al., 2011; Mitchell, 2011). Multiple prefrontal regions including OFC, vmPFC, dmPFC, vlPFC, and dlPFC are also associated with emotion regulation (Dolcos et al., 2011; Mitchell, 2011; Golkar et al., 2012).

In the work described here, we performed a new analysis of fMRI data previously presented by Brown et al. (2012). The original Brown et al. (2012) paper did not consider participant risk tendencies nor impulsivity levels. The current work features a new analysis of relationships between individual risk behavior tendencies or impulsivity scores and fMRI activation patterns in the emotional Go/NoGo task. The analysis presented here is statistically independent of the previous analysis presented in Brown et al. (2012).

Brown et al. (2012) found fMRI changes related to response-inhibition and emotion processing in many brain regions. In the response inhibition contrast, they found significantly larger Go vs. NoGo activation in left motor cortex and other regions and larger NoGo vs. Go activation in ventrolateral prefrontal cortex as well as other cortical regions. These findings are consistent with previous Go/NoGo studies (Garavan et al., 1999, 2002; Watanabe et al., 2002; Mostofsky et al., 2003; Aron et al., 2004b; Fassbender et al., 2004; Kelly et al., 2004; Rubia et al., 2005a; Wager et al., 2005; Aron et al., 2007; Mitchell, 2011). In the emotional valence contrast, they found greater activation for aversive vs. neutral distractor pictures in orbitofrontal cortex, lateral prefrontal cortex, insula, the amygdala and surrounding cortex, anterior cingulate cortex, medial prefrontal cortex, and bilateral posterior middle temporal gyrus and angular gyrus. These results are also consistent with studies of emotional picture processing (Irwin et al., 1996; Bermpohl et al., 2006; Meseguer et al., 2007). Brown et al. (2012) looked specifically at interaction of fMRI activation patterns related to response-inhibition and emotion processing in vlPFC. They found that these two sources of fMRI activation changes summated in a straight-forward manner; emotional context (aversive vs. neutral distractors) did not suppress or potentiate fMRI signals related to response inhibition in vlPFC (see Brown et al., 2012 for further details).

In the current study, we tested several hypotheses. We expected fMRI activation patterns evoked in prefrontal regions by the response inhibition and emotion processing components of the emotional Go/NoGo task to exhibit modulation based on participants' high-risk tendencies and impulsivity levels. Based on Horn et al. (2003) and Asahi et al. (2004), we expected impulse-control activation in right dlPFC and dmPFC to be modulated by psychometric impulsivity scores. We did exploratory analyses looking for other prefrontal regions that might show modulation of fMRI activation related to response inhibition and/or emotional stimulus processing based on participants' risk tendencies and impulsivity scores. Given the previously-suggested role of impulsivity in contributing to high-risk behavior (see discussion above), we expected this exploratory analysis to reveal a subset of prefrontal brain regions exhibiting similar modulation of fMRI activity patterns by both risk tendency and impulsivity. In addition, given that aversive emotional contexts have been suggested to promote impulsive decision-making to escape aversive stimuli or circumstances in certain individuals (see negative urgency as discussed in Whiteside and Lynam, 2001; Cyders and Smith, 2008), we expected that the exploratory analysis might reveal a relationship between participant risk behavior or impulsivity tendencies and fMRI activation patterns related to response inhibition in the presence of aversive distractors.

## 2. Methods

The Health Research Ethics Board at the University of Alberta approved this study.

### 2.1. Participants

Nineteen young adults were recruited into the study (12 female and 7 male, age range 18–28 years, mean age 22.7 ± 2.3 years). All participants were undergraduate or graduate students recruited from the University of Alberta student population. Based on the Edinburgh Handedness Inventory (Oldfield, 1971), 16 participants were right-handed. One participant was left-handed, and two were ambidextrous. All participants gave informed, written consent in English. Participants reported no history of diagnosed psychiatric or neurological disorder and no history of learning disability. Participants exhibited low to moderate risk behavior tendencies based on the CARE risk questionnaire. Based on the BIS impulsivity questionnaire, participants fell in the low to moderate impulsivity range. No participants exhibited very high risk behavior or very high impulsivity tendencie (see Sections 2.2, 3.1 for details of CARE and BIS scores).

### 2.2. Questionnaires

We used the expected involvement component of the Cognitive Appraisal of Risky Events (CARE) questionnaire (Fromme et al., 1997) to assess each participant's risk behavior tendencies. This instrument includes 30 questions that ask a participant to rate how likely they are to engage in various risk-related behaviors in the next 6 months. A seven-point Likert scale is used with ratings ranging from 1 (not at all likely) to 7 (extremely likely). The CARE provides six risk behavior subscores: illegal drug use, fighting and petty crime, high-risk sex, alcohol abuse, high-risk sports, and cheating at or neglect of academic/employment work. To derive a single risk score for each participant, we took the mean score over the six subscores. This overall CARE risk score could range from a minimum of 1 (lowest risk tendency) to a maximum of 7 (highest risk tendency).

To assess participants' impulsivity, we used the Barratt Impulsivity Scale, version 11 (BIS) (Patton et al., 1995). This questionnaire includes 30 questions that assess a participant's frequency of engaging in impulsive or non-impulsive activities and mental states. Assessment is on a four-point scale with the values (1) rarely/never, (2) occasionally, (3) often, and (4) almost always/always. The BIS includes six first order subscales: attentional, cognitive instability, motor, perseverance, self-control, and cognitive complexity. We took the sum over all 30 questions (after reversing scores for appropriate items) as a participant's impulsivity score. This is equivalent to taking the sum of the six first order subscale scores. Overall BIS scores can range from 30 (least impulsive) to 120 (most impulsive). BIS scores from 52 to 71 represent a normal range of impulsivity, with scores at or below 51 indicating a very controlled, non-impulsive individual and scores at or above 72 representing a highly-impulsive individual (Stanford et al., 2009).

### 2.3. Task

**Figure 1:**
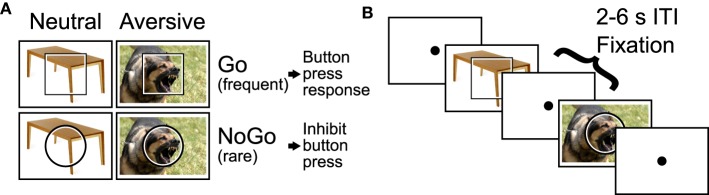
**Emotional Go/NoGo task. (A)** Each trial was either a Go or NoGo trial and featured an motionally neutral or aversive distractor picture. **(B)** Example segment of two trials with 2–6 s fixation intertrial intervals (ITIs) interleaved.

We employed an emotional Go/NoGo task (see Donders, 1868/1969; Hester and Garavan, 2004), which presented emotional distractor pictures simultaneously with the Go and NoGo stimuli. In each trial, the participant was shown a square or circle, lasting 2 s, which served as the Go or NoGo stimulus (see Figure 1). The assignment of shape to trial type was counterbalanced across participants. Each Go or NoGo stimulus was superimposed on a task-irrelevant distractor image. Each distractor image was either emotionally neutral or aversive. Distractor images were taken from the International Affective Pictures System (IAPS; Lang et al., 2008). On Go trials, the participant had to press a button with their right index finger. On NoGo trials, the participant had to withold the button press response. To make the Go response more automatic (prepotent), Go and NoGo trials were presented at a 4:1 ratio. The task included four trial types: neutral Go, neutral NoGo, aversive Go, and aversive NoGo. Between trials, participants fixated a dot located at screen center (Figure 1B).

IAPS images were chosen as follows. IAPS images were screened by two child psychiatrists to be acceptable for use with our participant population and with adolescent psychiatric participants in a concurrent study (Brown et al., under review). From the screened images, aversive and neutral distractor pictures were selected based on the IAPS measures of valence and arousal from the normative sample reported in Lang et al. (2008). To maximize the effect of distractor valence, we used image selection criteria that created two non-overlapping clusters of images in two-dimensional arousal-valence space, one cluster for aversive distractors and one for neutral distractors (see Supplementary Figure 1). Specifically, we selected the 100 aversive IAPS images that had valence ratings ≤ 3.6 and were closest to [arousal, valence] target position [9, 1]. Position [9, 1] represents the most aversive (lowest valence rating), most arousing possible score. We selected the 104 neutral images with valence ratings > 3.6 and < 6.4 that were closest to [arousal, valence] target position [1, 5], which represents a neutral valence and the smallest possible arousal score. It would have been preferable to match distractor images for scene complexity, number of objects, and so on across the different trial types. Unfortunately, the IAPS set did not include enough images to permit such matching while also satisfying the above-described criteria, namely, screening by psychiatrists and separation into two non-overlapping clusters (as can be seen in Supplementary Figure 1). Aversive distractor pictures presented a variety of scenes including threatening animals, aggressive human faces, individuals wielding guns in a threatening manner, human injuries, surgical scenes, vehicle accidents, terrorism-related scenes, individuals vomiting, and dirty toilets including feces.

Trials were presented in a rapid event-related design. Each Go or NoGo trial lasted one volume, i.e., 2 s. Inter-trial intervals were pseudo-randomized from the set {2, 4, 6 s}, distributed 30% 2 s, 40% 4 s, 30% 6 s with a mean of 4 s. Trial sequences and timings were derived using custom Python code to ensure linear independence of trial activations (see Burock et al., 1998). First-order counterbalancing of trial sequences was used to avoid first-order interaction effects between adjacent trials. To avoid interaction of BOLD non-linearity with inter-trial intervals and trial types, each of the four trial types was preceded in equal proportions by the 2, 4, and 6 s inter-trial intervals. Participants each completed four 330 s functional runs with a combined total of 204 trials including 84 neutral Go trials, 80 aversive Go trials, 20 neutral NoGo trials, and 20 aversive NoGo trials. The first trial of every run was always a neutral Go trial.

### 2.4. IAPS distractor picture ratings

After completing the fMRI scanning component of the study, participants rated the 204 IAPS distractor images used in the emotional Go/NoGo task for valence and arousal using the 9-point Likert scale (range 1–9) described in Lang et al. (2008). Valence ratings indicate participants' judgements of how pleasant or unpleasant a picture is (1: most unpleasant, 5: neutral, 9: most pleasant). Arousal ratings indicate how exciting or not exciting a picture is (1: not at all exciting, 5: neutral, 9: most exciting).

### 2.5. Analysis of questionnaires, IAPS ratings, and task performance

We computed a participant's overall CARE risk score as the mean of the six CARE subscales (see above and Fromme et al., 1997). To assess relative contributions of each subscale to the overall risk score, we did a separate correlation analysis of scores from each subscale vs. the overall CARE score. A participant's overall Barratt impulsivity score was computed as the sum across all questionnaire items (see above and Patton et al., 1995). We did a separate correlation analysis of scores from each of the six BIS first order subscales vs. the overall BIS score. Finally, we did a correlation analysis of overall CARE risk vs. BIS impulsivity scores. Two-tailed *t*-tests were used to test whether each of the above correlations was significantly different from zero. Kolmogorov-Smirnov tests were used to check for non-normality in the distributions of participants' CARE and BIS scores.

Valence and arousal ratings for IAPS distractor pictures were analyzed with separate mixed effects ANOVAs in the R statistical language using the within-subject factors Response Inhibition (Go vs. NoGo) and Valence (aversive vs. neutral). We found differences in valence and arousal ratings for aversive vs. neutral distractor images but not between images used in Go vs. NoGo trials (see Section 3.2). A subsequent analysis examined relationships between valence and arousal ratings and CARE risk and BIS impulsivity scores. After collapsing across Go vs. NoGo trials, each participant's average rating for aversive distractor images and for neutral distractor images was computed, as was the difference between these two (aversive−neutral distractor ratings). We then computed the correlations of these differences vs. CARE risk scores and vs. BIS impulsivity scores. Two-tailed *t*-tests were used to test whether these correlations were significantly different from zero.

Behavioral data from task performance (error rates and latencies) were analyzed as follows. A Kolmogorov-Smirnov test was used to test for non-normality in the distribution of commission error rates for NoGo trials (collapsed across distractor valence). This test did not indicate a significant difference from normality (see Section 3.3). Commission error rates on NoGo trials (collapsed across distractor valence) were compared against zero with a one-tailed *t*-test. Kolmogorov-Smirnov tests for commission error rates for neutral NoGo trials and aversive NoGo trials—considered as separate sets of data, not collapsed across neutral and aversive distractors—did indicate that the distributions were non-Gaussian (see Section 3.3). Therefore, error rates for NoGo trials with neutral vs. aversive distractors were compared using permutation testing. Omission errors on Go trials were very rare with 17 of 19 participants making no such errors and the other two participants making very few commission errors (4.3% and 0.6%, see Section 3.3). Therefore, we simply reported the Go trial omission error rates without doing statistical comparisons. Latencies for Go trials with neutral vs. aversive distractor pictures were compared using a two-tailed *t*-test. Kolmogorov-Smirnov tests were used to test normality in the distributions of neutral Go and aversive Go trial latencies. These tests did not indicate significant deviation from normality (see Section 3.3). We tested for relationships between NoGo trial error rates (collapsing across neutral vs. aversive distractors) and CARE risk scores, as well as BIS impulsivity scores, using separate linear regression models and associated two-tailed *t*-tests. Similarly, we tested Go trial latencies (collapsing across neutral vs. aversive distractors) against CARE and BIS scores using separate linear regression models and associated two-tailed *t*-tests.

### 2.6. MRI scanning

Magnetic resonance imaging was done on the 4.7 Tesla Varian Inova scanner at the Peter S. Allen MR Research Center at the University of Alberta. We acquired blood oxygenation level dependent (BOLD) fMRI images with a T2^*^-weighted echo planar imaging sequence using these parameters: volume time 2.0 s, single shot, repeat time 2.0 s, echo time 19.0 ms, 3.0 mm isotropic voxels, 80 × 80 matrix, 240 × 240 mm^2^ field of view, 3.0 mm slice thickness, 36 axial slices, 108 mm through-plane coverage, interleaved slice collection order. We used 80% partial k-space in the phase encode direction (anterior-posterior). The fMRI scanning volume covered the entire cerebral cortex except for the ventral-posterior tip of occipital cortex in participants with larger heads. A high resolution T1-weighted structural scan was also acquired for each participant. This scan utilized a magnetization-prepared rapid acquisition gradient echo (MPRAGE) sequence with parameters: TR 9.4 ms, inversion time 300.0 ms, relaxation delay time (after readout prior to inversion) 300.0 ms, linear phase encoding, TE 3.7 ms, matrix 240 × 192 × 128, field of view 240 × 192 × 192 mm^3^, 1.0 × 1.0 × 1.5 mm^3^ voxels, whole brain coverage.

### 2.7. fMRI analysis

SPM8 and in-house MATLAB code were used for preprocessing of fMRI data. The preprocessing steps for each participant included: (1) 6 parameter rigid body motion correction of fMRI volumes in SPM8, (2) coregistration of fMRI data to MPRAGE anatomical scan in SPM8, (3) non-linear spatial warping (estimation and interpolation) of MPRAGE anatomical volume to MNI T1 template space at 1 × 1 × 1 mm resolution in SPM8, (4) interpolation of fMRI volumes into the T1 template space at 3 × 3 × 3 mm spatial resolution using warping parameters from step (3), (5) 8 mm full width at half maximum (FWHM) Gaussian spatial smoothing of fMRI volumes in SPM8.

Statistical modeling of fMRI data was done in two steps using custom-built MATLAB code. We first performed separate first-level, within-subjects general linear model (GLM) analyses on each participant. Within-subjects results were then combined using two different between-subjects mixed-effects analyses (Worsley et al., 2002). Each within-subject GLM included either four or five sets of finite impulse response (FIR) predictors, one set for each of the four trial types (neutral Go, aversive Go, neutral NoGo, aversive NoGo) and, for participants who made errors, one set of predictors for error trials (collapsed across trial types). Error trials were rare (0–10% of trials, per subject). The GLM included 10 FIR impulse predictors (corresponding to 10 functional volumes) per trial type. The FIR predictors represented deconvolved activation timecourses for the different trial types (see Serences, 2004). The GLM also included a set of nuisance predictors for each run consisting of constant run offset, linear drift, cosine, and sine with period equal to twice the run length, 6 rigid body motion parameters, and 6 impulses at the start of each run for spin saturation. We used a manually-constructed mask that excluded voxels outside the brain. The mask included 79,044 voxels (size 3 × 3 × 3 mm) inside the brain. Each within-subject GLM was fit to the data using weighted least squares that corrected for autocorrelated noise. Specifically, 10 autocorrelation coefficients (lags of 1–10 volume times) were computed for each functional slice across the whole brain using the residuals from a non-corrected initial GLM fit. Then, the design matrix and each voxel's timecourse were pre-whitened, and auto-correlation-corrected beta weights were computed as described in Burock and Dale (2000) and Worsley et al. (2002). For each subject separately, we computed three first-level (within-subjects) statistical contrast maps (two-tailed t statistic maps) from the GLM beta weights. The response inhibition contrast was (aversive NoGo + neutral NoGo) − (aversive Go + neutral Go). The emotional valence contrast was (aversive NoGo + aversive Go) − (neutral NoGo + neutral Go). The emotional response inhibition contrast was (aversive NoGo − aversive Go). Contrasts were computed from the FIR beta weights representing activation across the 3rd and 4th time points of the FIR deconvolved timecourses. The 3rd and 4th time points, which correspond to 4 and 6 s from trial start, were chosen *a priori* based on the typical BOLD hemodynamic peak time around 4–6 s (Aguirre et al., 1998).

For each of the three first-level statistical contrasts, we performed second-level analyses combining results across participants and testing for significant relationships between first level contrast values and CARE risk scores and/or BIS impulsivity scores. One goal was to identify brain regions that showed a relationship between a first level contrast and one of the questionnaires (either CARE or BIS scores) but not the other one. One incorrect approach would have been to do second level regressions against a given questionnaire (e.g., CARE scores), identify significant regions of interest (ROIs), and perform follow-up F-tests comparing regression against CARE vs. BIS scores on each ROI. This approach would have created dangers from double-dipping (Kriegeskorte et al., 2009; Vul et al., 2009). Instead, we used the approach described below.

For each of the three first-level statistical contrasts, we performed three second-level, mixed-effects analyses combining results across participants. The first analysis tested for significant relationships with CARE scores where CARE scores accounted for significantly more variance in first level contrast values than BIS scores (CARE > BIS), assessed using *F*-tests. The second analysis tested for significant relationships with BIS scores where BIS scores accounted for significantly more variance than CARE scores (BIS > CARE), assessed using *F*-tests. The third analysis tested for significant relationships with both CARE and BIS scores (conjunction analysis). Specific computational details for the second-level analyses are provided in Supplementary Methods Section 1.1. In total, there were nine second-level statistical maps:

- **Map 1:** response inhibition contrast vs. CARE scores with CARE > BIS,
- **Map 2:** response inhibition contrast vs. BIS scores with BIS > CARE,
- **Map 3:** response inhibition contrast vs. CARE AND BIS scores,
- **Map 4:** emotional valence contrast vs. CARE scores with CARE > BIS,
- **Map 5:** emotional valence contrast vs. BIS scores with BIS > CARE,
- **Map 6:** emotional valence contrast vs. CARE AND BIS scores.
- **Map 7:** emotional response inhibition contrast vs. CARE scores with CARE > BIS,
- **Map 8:** emotional response inhibition contrast vs. BIS scores with BIS > CARE,
- **Map 9:** emotional response inhibition contrast vs. CARE AND BIS scores.

Statistical t-maps were thresholded voxelwise at *p* < 0.05. A cluster mass threshold of 465 was also applied to each map to correct for multiple comparisons at *p* < 0.05 across the voxel population as well as the nine second-level statistical comparisons. Cluster mass is the sum of absolute *t*-values from all voxels in the cluster. The cluster mass threshold was determined using Monte Carlo simulation based on the method of AlphaSim (Ward, 2000), modified to account for comparisons across all nine second-level maps. Statistical results were visualized using MATLAB and EasyFMRI (www.easyfmri.com).

We did follow-up quality assurance analyses on the nine second-level maps described above. None of the three conjunction maps (Maps 3, 6, and 9) revealed any significant regions surviving multiple comparison correction. For the other second-level maps, each of which did reveal one or more significant voxel clusters, an automated algorithm was used to grow a cluster of voxels around each positive or negative statistical peak (local extremum) in the associated t-map (after filtering via an F-map, if appropriate, see Supplementary Methods Section 1.1). In some cases, two or more of the resulting clusters fell within the same anatomical structure. In these cases, we combined those clusters. There were 36 such clusters in total from Maps 1, 2, 4, 5, 7, and 8. For a given cluster, each participant's mean BOLD signal was computed by averaging across all voxels in the cluster. First-level GLM analyses were then conducted on the average timecourses, followed by second-level regression against CARE or BIS scores. Event-related activation timecourses for each of the four trial types were derived from the first-level finite impulse response models and averaged across participants. We discarded two clusters whose activation timecourses were severely dissimilar to the expected difference of gammas hemodynamic response function shape (see Huettel et al., 2008, ch. 7), as determined by visual inspection. In some clusters, statistical significance in the second-level analyses was dependent on one or two outlier participants. We re-ran all second-level analyses excluding the two most extreme participants, either the participants with the smallest and largest fMRI contrast values for the given cluster or the participants with the smallest and largest CARE scores or BIS scores, as appropriate. Clusters that failed to reach significance without these two participants were discarded. 23 of the 36 clusters were discarded in this way. We discarded a total of 25 clusters based on the above quality assurance criteria, and we present results only for the 11 remaining clusters.

For exploratory purposes, we computed correlations between significant regions identified in Maps 1–9 and participant scores on the CARE and BIS subscales (see Supplementary Methods Section 1.2 for details).

## 3. Results

### 3.1. Risk and impulsivity scores

Participant CARE risk scores had a mean of 2.69 ± 0.58 and ranged from 1.66 to 4.06. That is, the participants in this study exhibited low to medium risk tendencies based on the CARE questionnaire, with no participants exhibiting very high-risk tendencies. The distribution of CARE scores did not differ significantly from the Gaussian (*p* = 0.90, Kolmogorov-Smirnov test). Differences in participants' CARE risk scores were driven primarily by differences in the heavy drinking CARE subscale, as well as by differences in drug use, aggression, and academic/work subscales (see Table 1).

**Table 1:** **Summary of CARE scores and subscale scores**.

	**Mean ± Std**	**Min**	**Max**	***R***	***P***
CARE overall score (mean of subscales)	2.69 ± 0.58	1.66	4.06	–	–
CARE illicit drug use	2.33 ± 1.53	1.00	6.67	0.54	0.017
CARE aggressive / illegal behaviors	1.63 ± 0.49	1.00	2.56	0.53	0.021
CARE risky sexual activities	1.73 ± 1.00	1.00	5.17	0.18	0.46
CARE heavy drinking	3.75 ± 1.84	1.00	6.67	0.79	6.5 × 10^−5^
CARE high risk sports	4.00 ± 1.62	2.00	7.00	0.22	0.36
CARE academic/work behaviors	2.69 ± 0.89	1.00	4.20	0.49	0.032

R denotes correlation coefficient comparing subscore against CARE score (mean of subscores). P indicates p-value from t-test comparing correlation against zero with *df* = 17.

Participants' Barratt impulsivity scale (BIS) scores had a mean of 58.11 ± 8.14 with a range of 43–71. That is, participants ranged from non-impulsive to moderately-impulsive. No participants fell in the highly-impulsive range based on Stanford et al. (2009)'s criterion (BIS score ≥ 72). The distribution of BIS scores did not differ significantly from the Gaussian (*p* = 0.39, Kolmogorov-Smirnov test). Differences in overall BIS scores were driven most strongly by differences in the BIS self-control, cognitive instability, and attentional subscales, while differences in the motor and perseverance subscales also contributed (see Table 2).

**Table 2:** **Summary of BIS scores and subscale scores**.

	**Mean ± Std**	**Min**	**Max**	***R***	***P***
BIS overall score (sum of subscales)	58.11 ± 8.14	43	71	–	–
BIS 1st order attentional subscale	9.53 ± 2.82	5	14	0.72	0.00055
BIS 1st order cognitive instability subscale	6.32 ± 2.00	3	9	0.75	0.00023
BIS 1st order motor subscale	14.95 ± 1.99	11	18	0.68	0.0014
BIS 1st order perseverance subscale	6.79 ± 1.36	4	9	0.48	0.038
BIS 1st order self-control subscale	10.58 ± 2.55	6	15	0.80	4.7 × 10^−5^
BIS 1st order cognitive complexity subscale	9.95 ± 2.17	5	14	0.28	0.25

R denotes correlation coefficient comparing subscore against overall BIS score (sum of 1st order subscores). P indicates p-value from t-test comparing correlation against zero with *df* = 17.

**Figure 2:**
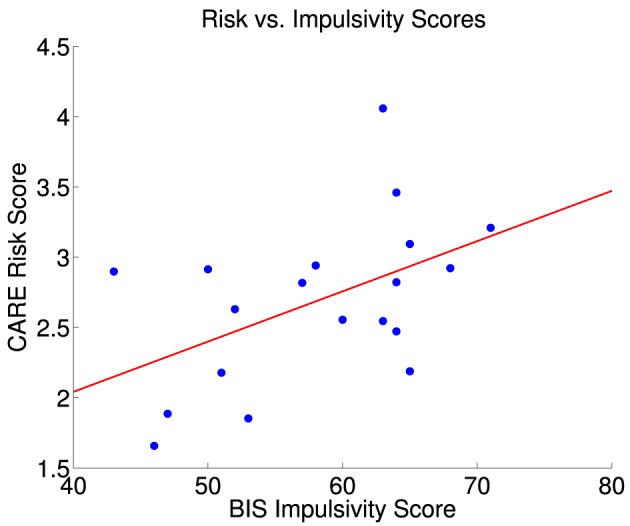
**CARE risk scores vs. BIS impulsivity scores for 19 participants**. The red line is the best fit linear regression of CARE scores against BIS scores. Correlation between BIS and CARE scores was 0.50 (significant, *p* = 0.030, *t* = 2.36, *df* = 17). BIS scores explained 20.3% of the variance in the CARE scores. The largest statistical influence (Cook's distance D) across all data point was 0.487.

There was a partial linear relationship between participant BIS and CARE scores (Figure 2). The correlation coefficient between BIS and CARE scores was 0.50, which was significantly different from zero (*p* = 0.030, *t* = 2.36, *df* = 17). BIS scores explained only 20.3% of the variance in the CARE scores.

### 3.2. Distractor picture ratings

Participants rated IAPS distractor pictures for valence and arousal on a 9-point Likert scale (see Section 2.4). Mean valence rating for aversive distractors was 3.18 ± 0.64 (mean ± std). Mean valence rating for neutral distractors was 5.32 ± 0.21. Valence ratings were significantly lower for aversive distractors (mixed effects ANOVA, *p* < 1.0 × 10^−10^, *F* = 175.3, *df* = 1, 18). There were no significant differences in valence scores for distractors on Go vs. NoGo trials (*p* = 0.58, *F* = 0.31, *df* = 1, 18), nor was there a significant interaction effect of Go vs. NoGo × aversive vs. neutral (*p* = 0.68, *F* = 0.18, *df* = 1, 18). Arousal ratings were significantly higher for aversive distractor trials (4.94 ± 1.19) compared to neutral distractor trials (2.10 ± 0.92). The main effect of aversive vs. neutral distractors on arousal ratings was significant (mixed effects ANOVA, *p* = 1.3 × 10^−9^, *F* = 128.3, *df* = 1, 18). The main effect on arousal for Go vs. NoGo trials was not significant (*p* = 0.90, *F* = 0.016, *df* = 1, 18), nor was the interaction effect significant (*p* = 0.063, *F* = 3.92, *df* = 1, 18).

**Figure 3:**
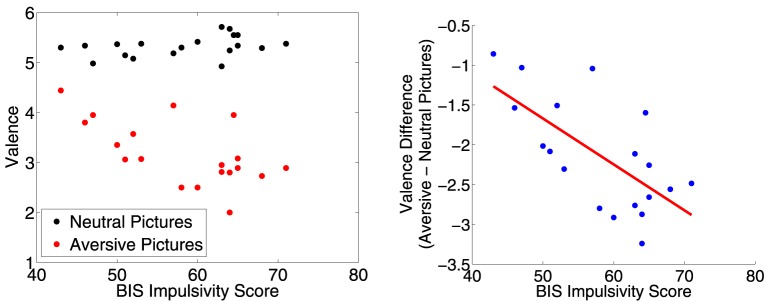
**Left:** Participants' mean valence ratings for neutral and aversive distractor pictures plotted against their BIS impulsivity scores. **Right**: Difference in mean valence scores (aversive−neutral pictures) plotted against BIS impulsivity scores. Red line is best fit linear regression line. More impulsive participants rated the aversive pictures as more unpleasant (lower valence score). The correlation was significant (*r* = −0.67, *p* = 0.002, *t* = −3.76, *df* = 17).

We did separate correlation analyses of CARE risk scores and Barratt impulsivity scores against the difference between ratings for aversive vs. neutral pictures. (See Section 2.5 for analysis details.) The correlation between CARE risk scores and the difference in valence scores (aversive−neutral pictures) was not significantly different from zero (*r* = −0.40, *p* = 0.090, *t* = −1.80, *df* = 17), nor was the correlation between CARE risk scores and the difference in arousal scores (*r* = 0.35, *p* = 0.14, *t* = 1.54, *df* = 17). The correlation between BIS impulsivity scores and the difference in valence scores was significantly different from zero (*r* = −0.67, *p* = 0.002, *t* = −3.76, *df* = 17). More impulsive participants rated the aversive pictures as more unpleasant (lower valence score) as shown in Figure 3. The correlation between BIS impulsivity scores and the difference in arousal scores was not significantly different from zero (*r* = 0.32, *p* = 0.18, *t* = 1.40, *df* = 17).

### 3.3. Task performance

Participants made commission errors on NoGo trials (collapsed across distractor valence) at a mean rate of 3.7 ± 3.8%, which was low but significantly above zero (*p* = 0.00022, *t* = 4.3, *df* = 18, one-tailed *t*-test). The distribution of participants' error rates for NoGo trials (collapsed across distractor valence) was not significantly different from the Gaussian (*p* = 0.35, Kolmogorov-Smirnov test). Without collapsing across distractor valence, the distribution of neutral NoGo trial error rates approached significant difference from the Gaussian (*p* = 0.057, Kolmogorov-Smirnov test), and aversive NoGo trial error rates were significantly non-Gaussian (*p* = 0.039, Kolmogorov-Smirnov test). Error rates did not differ significantly between NoGo trials with aversive vs. neutral distractors (*p* = 0.56, permutation test). Seventeen of nineteen participants made no omission errors on Go trials, while the other two participants had low omission error rates of 4.3% and 0.6%. Go trial latencies were 616 ± 135 ms with neutral distractors and 631 ± 148 ms with aversive distractors. The distributions of neutral Go and aversive Go trial latencies did not differ significantly from the Gaussian (respectively, *p* = 0.95 and *p* = 0.98, Kolmogorov-Smirnov tests). The difference between neutral Go and aversive Go trial latencies was significant (*p* = 0.033, *t* = 2.3, *df* = 18, two-tailed *t*-test). NoGo error rates and Go trial latencies did not show any significant relationships with either CARE risk scores or Barratt impulsivity scores on linear regression tests (*p* > 0.5, |*t*| < 0.69, *df* = 17). Similarly, Horn et al. (2003) and Asahi et al. (2004) did not find significant relationships between participant impulsivity scores and Go/NoGo task performance.

### 3.4. fMRI results independent of risk and impulsivity scores

Brown et al. (2012) previously presented an analysis of fMRI activation related to response inhibition and distractor picture valence in the fMRI dataset used in the current study. Here, we excluded one participant from Brown et al. (2012)'s analysis, as this person did not complete the BIS. This exclusion did not significantly change the results as presented in Brown et al. (2012). Briefly, in the response inhibition contrast, we found significantly larger Go vs. NoGo activation in left motor cortex and other regions and larger NoGo vs. Go activation in ventrolateral prefrontal cortex as well as other cortical regions (see Supplementary Figure 2). In the emotional valence contrast, we found greater activation for aversive vs. neutral distractor pictures in orbitofrontal cortex, lateral prefrontal cortex, insula, the amygdala and surrounding cortex, anterior cingulate cortex, medial prefrontal cortex, and bilateral posterior middle temporal gyrus and angular gyrus (see Supplementary Figure 3) (See Brown et al., 2012 for further details). The emotional response inhibition contrast (aversive NoGo−aversive Go), also revealed large regions of significant difference in all major lobes of the brain, including regions in dlPFC, vlPFC, right anterior insula, and right OFC showing greater activation for aversive NoGo trials (see Supplementary Figure 4). There was a right side laterality in that the right vlPFC and right dlPFC clusters were larger than the left ones.

### 3.5. fMRI response inhibition contrast vs. risk and impulsivity scores

**Figure 4:**
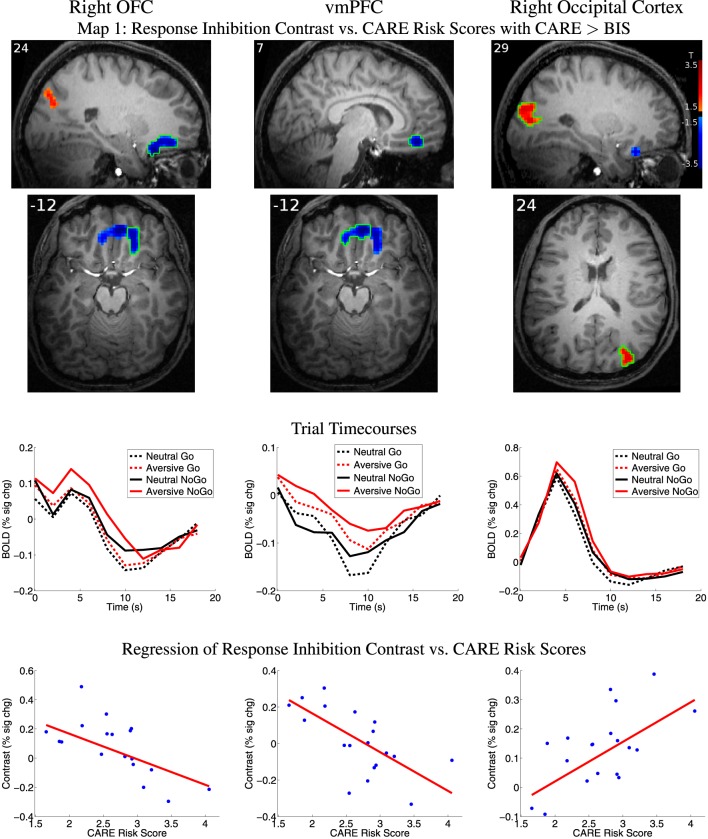
**Top two rows:** Map 1 statistical t-map for regression of CARE risk scores against fMRI response inhibition contrast (NoGo−Go) where CARE scores also accounted for significantly more variance than BIS scores. Red and blue regions, respectively, exhibited larger contrast magnitudes in participants with higher and lower CARE risk scores. All results *p* < 0.05 (corrected for multiple comparisons). Color bar in right-most image indicates *t*-value scaling. Slice X- or Z-coordinate in MNI space shown in upper-left. Axial images' left side corresponds to left side of brain. OFC: orbitofrontal cortex. vmPFC: ventromedial prefrontal cortex. **Third row:** Mean deconvolved event-related timecourses for four trial types for regions outlined in green in first row. **Fourth row:** Scatter plots of response inhibition contrast magnitude vs. participants' CARE risk scores for regions outlined in green in first row. Red line shows linear regression of contrast magnitude against participant risk scores. Maximum values for participants' statistical influence (Cook's distance) on linear regression results were: right OFC 0.295, vmPFC 0.760, right occipital 0.247 (see Supplementary Table 1).

We examined relationships between the response inhibition contrast (NoGo−Go) and CARE risk scores and between the inhibition contrast and BIS impulsivity scores (see Section 2.7 for methodological details). Statistical Map 1 tested regression of response inhibition contrast against CARE scores where CARE scores also accounted for significantly more variance than BIS scores (see Section 2.7). Map 1 revealed significant inverse relationships (*p* < 0.05, corrected) in a large cluster with two foci, one in right orbitofrontal cortex (OFC) and the other in ventromedial prefrontal cortex (vmPFC) (See Figure 4 and Table 3). As required by Map 1's inclusion criteria, CARE scores accounted for significantly more variance in the inhibition contrast values than did BIS scores in these regions (also see Table 3). These regions did not show a significant relationship between inhibition contrast and BIS scores (also see Table 3). The right OFC region included voxels in the medial orbital gyrus, rostral and caudal parts of the medial orbital sulcus, and medial portions of the anterior and posterior orbital gyri (based on Chiavaras and Petrides (2000)'s description of orbitofrontal anatomy). The vmPFC cluster was bilateral, centered supero-inferiorly on the suborbital sulcus, and included voxels in the adjacent ventromedial part of the medial aspect of the superior frontal gyrus as well as the medial aspect of the gyrus rectus. In the vmPFC region, the four trial types evoked deactivation of the BOLD signal (Figure 4 third row, middle panel). Specifically, in this region, participants with lower CARE risk scores exhibited larger negative BOLD deflections for Go vs. NoGo trials (resulting in positive NoGo-Go contrast values), while higher CARE score participants exhibited larger negative BOLD deflections for NoGo vs. Go trials (resulting in negative NoGo-Go contrast values). Also see scatterplot, middle of bottom row, Figure 4. Map 1 indicated that there was also a significant positive relationship between response inhibition contrast and CARE scores in right occipital cortex (Figure 4, Table 3). Measures of influence (Cook's distance) were below 1 for all participants for all of the above regions (see Supplementary Table 1). Correlation analyses revealed significant relationships, in these regions, between response inhibition contrast and several CARE subscales (see Supplementary Table 2).

**Table 3:** **Summary of significant regions from Map 1**.

**Region**	***X***	***Y***	***Z***	**Volume**	**Regression vs. CARE**		**Regression vs. BIS**		***F*-test**	
	**(mm)**			**(mm^3^)**	***P***	***T***	***P***	***T***	***P***	***F***
**From Map 1: Response Inhibition Contrast vs CARE Scores with CARE > BIS**									***F*-test CARE > BIS**	
Right OFC	27.0	26.0	−20.0	3537	0.0155	−2.46	0.1334	−1.51	<0.0001	19.05
vmPFC	9.0	47.0	−11.0	4158	0.0192	−2.38	0.4458	−0.77	<0.0001	28.42
Right occipital	33.0	−85.0	22.0	4401	0.0261	2.26	0.3170	1.01	<0.0001	22.75

Summary of significant regions from Map 1: comparison of fMRI response inhibition contrast (NoGo—Go) vs. CARE risk scores where CARE scores also accounted for significantly more variance than BIS scores. Statistical comparison of inhibition contrast vs. BIS impulsivity scores (Map 2) did not yield any significant regions. X, Y, Z: MNI coordinates of region's peak statistical voxel. P- and t-values are median values across all voxels in a region (*df* = 98). Positive and negative t-values indicate, respectively, greater and lesser inhibition contrast values for participants with larger CARE scores. F-test CARE > BIS are median values across each region from the F-map testing whether CARE scores accounted for significantly more variance in response inhibition contrast values than BIS scores (*df* = 1, 97). See Section 2.7 for analysis details. OFC: orbitofrontal cortex. vmPFC: bilateral ventromedial prefrontal cortex. Occipital: occipital cortex.

The statistical comparison of response inhibition contrast vs. BIS scores (Map 2, see Section 2.7) did not reveal any significant regions after correction for multiple comparisons and quality assurance exclusions. The conjunction analysis of regressions of response inhibition contrast against CARE and against BIS scores (Map 3, see Section 2.7) revealed no significant clusters surviving multiple comparison correction.

### 3.6. fMRI emotional valence contrast vs. risk and impulsivity scores

We examined relationships between the emotional valence contrast (aversive−neutral distractor pictures) vs. CARE risk scores and vs. BIS impulsivity scores. See Section 2.7 for methodological details.

Map 4 (see Section 2.7) revealed a positive relationship between emotional valence contrast amplitude and participants' CARE scores in right occipital cortex and dorsomedial cerebellum (Table 4). As required by Map 4's inclusion criteria, CARE scores accounted for significantly more variance in emotion contrast amplitudes compared to BIS scores (also see Table 4) in these regions. These regions did not exhibit significant relationships with BIS scores (also see Table 4). Measures of influence (Cook's distance) were below 1 for all participants for all of the above regions (see Supplementary Table 1). Correlation analyses revealed significant relationships between emotional valence contrast and several CARE subscales in these regions (see Supplementarypreviously presented an analysis of fMRI Table 3).

**Table 4:** **Summary of significant regions from Maps 4 and 5**.

**Region**	***X***	***Y***	***Z***	**Volume**	**Regression vs. CARE**		**Regression vs. BIS**		***F*-test**	
	**(mm)**			**(mm^3^)**	***P***	***T***	***P***	***T***	***P***	***F***
**From Map 4: Emotional Valence Contrast vs CARE Scores with CARE > BIS**									***F*-test CARE > BIS**	
Right occipital	27.0	−79.0	10.0	13365	0.0135	2.52	0.3657	0.91	<0.0001	21.07
dmCereb	3.0	−46.0	−2.0	13041	0.0254	2.27	0.7772	0.28	<0.0001	29.68
**From Map 5: Emotional Valence Contrast vs BIS Scores with BIS > CARE**									***F*-test BIS > CARE**	
dmPFC	0.0	44.0	43.0	20547	0.6023	−0.52	0.0190	−2.39	<0.0001	28.99
pgACC	−9.0	41.0	10.0	6912	0.2881	−1.07	0.0205	−2.35	<0.0001	25.55
Right pOFC	27.0	23.0	−20.0	1512	0.0470	−2.01	0.0208	−2.35	<0.0001	23.43
Right temp pole	30.0	26.0	−35.0	1296	0.0733	−1.81	0.0163	−2.44	<0.0001	16.08

Summary of significant regions from Maps 4 and 5. Map 4 tested comparison of fMRI emotional valence contrast (aversive - neutral distractors) vs. CARE scores where CARE scores also accounted for more variance than BIS scores. Map 5 tested regression of emotional valence contrast vs. BIS scores where BIS scores also accounted for more variance than CARE scores. X, Y, Z: MNI coordinates of region's peak statistical voxel. P- and t-values are median values across all voxels in each region (*df* = 98). F-test CARE > BIS values are median values across each region from the F-map testing whether CARE scores accounted for significantly more variance in emotional valence contrast values than BIS scores (*df* = 1, 97). F-test BIS > CARE shows median values from the F-map testing whether BIS scores accounted for significantly more variance in emotional valence contrast values than CARE scores (*df* = 1, 97). See Section 2.7 for details of analysis. Occipital: occipital cortex. dmCereb: dorsomedial cerebellum. dmPFC: dorsomedial prefrontal cortex. pgACC: perigenual anterior cingulate cortex. pOFC: posterior orbitofrontal cortex. Temp Pole: temporal pole.

**Figure 5:**
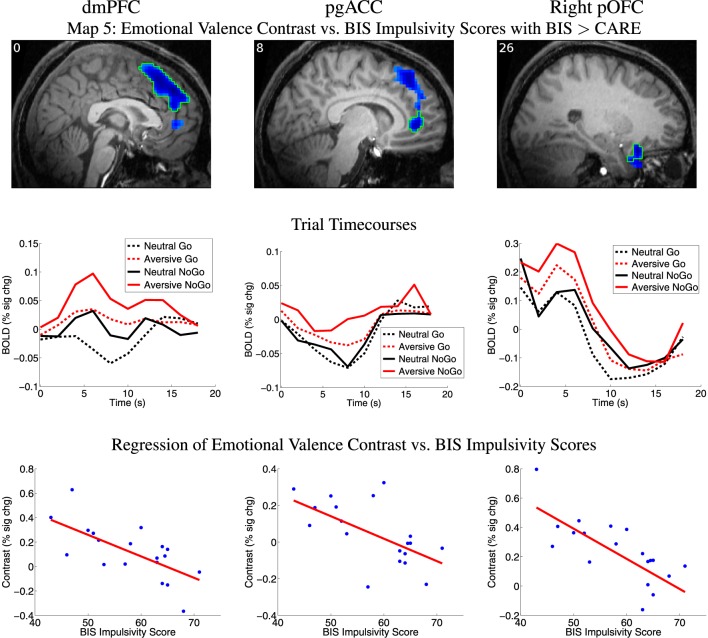
**Top row:** Map 5 statistical t-map for regression of BIS impulsivity scores against fMRI emotional valence contrast (aversive−neutral pictures) where BIS scores also accounted for significantly more variance than CARE scores. Blue regions exhibited smaller contrast magnitude in participants with higher BIS impulsivity scores. All results *p* < 0.05 (corrected for multiple comparisons). See Section 2.7 for details. *t*-value color scaling as in Figure 4. Slice X coordinate in MNI space shown in upper-left. pOFC: posterior orbitofrontal cortex. dmPFC: dorsomedial prefrontal cortex. pgACC: perigenual anterior cingulate cortex. pOFC: posterior orbitofrontal cortex. **Second row:** Mean deconvolved timecourses for four trial types for regions outlined in green in first row. **Third row:** Scatter plots of emotional valence contrast magnitude vs. participants' BIS impulsivity scores for regions outlined in green in first row. Red line shows linear regression of contrast magnitude against participant impulsivity scores. Maximum values for participants' statistical influence (Cook's distance) on linear regression results were: dmPFC 0.410, pgACC 0.172, right pOFC 0.726 (see Supplementary Table 1).

Map 5 (see Section 2.7) revealed a significant inverse relationship between emotional valence contrast amplitude and BIS impulsivity scores in a large cluster in dorsomedial prefrontal cortex (dmPFC) (See Figure 5, Table 4). This region included a large portion of the dorsomedial aspect of the superior frontal gyrus as well as adjacent anterior cingulate sulcus. There was a similar relationship in voxel clusters in perigenual anterior cingulate cortex (pgACC), right posterior orbitofrontal cortex (pOFC), and right temporal pole (Figure 5, Table 4). The pgACC region was bilateral, centered antero-posteriorly on the anterior cingulate sulcus rostral to the genu of the corpus callosum, and extended into the adjacent anterior cingulate gyrus and slightly into the posterior part of the medial aspect of the superior frontal gyrus. pgACC exhibited BOLD deactivation in response to the four trial types (Figure 5 second row, middle panel). In this region, participants with low BIS impulsivity scores exhibited larger negative BOLD deflections for neutral distractor trials, whereas participants with higher BIS scores exhibited greater negative BOLD deflections for aversive distractor trials (see scatterplot, middle of bottom row, Figure 5). The pOFC region was located in the posterior orbital gyrus. This region overlapped partially with the most posterior part of the OFC region from Map 1 (response inhibition contrast vs. CARE scores). The dmPFC and pgACC regions did not overlap with the vmPFC region from Map 1. BIS scores accounted for significantly more variance in the emotional valence contrast values compared to CARE scores in all of these regions, as required by Map 5's inclusion criteria (also see Table 4). Map 4 (regression of emotional valence contrast vs. CARE scores) did not include significant clusters in these regions. In terms of regression of emotional valence contrast vs. CARE scores, the median across voxels in the right pOFC region from Map 5 did exhibit a significant inverse relationship between emotional valence contrast values and CARE scores (*p* = 0.047, Table 4), but this region did not survive multiple comparison correction (cluster mass thresholding, see Section 2.7) in the t-map calculation for Map 4. Measures of influence (Cook's distance) were below 1 for all participants for all of the above regions (see Supplementary Table 1). Correlation analyses revealed significant relationships between emotional valence contrast and several BIS subscales in these regions (see Supplementary Table 4).

Map 6 (conjunction of t-maps for emotional valence contrast vs. CARE scores and vs. BIS scores) did not reveal any significant regions surviving correction for multiple comparisons.

### 3.7. fMRI emotional response inhibition contrast vs. risk and impulsivity scores

We examined relationships between the emotional response inhibition contrast (aversive NoGo−aversive Go trials) vs. CARE risk scores and vs. BIS impulsivity scores. See Section 2.7 for methodological details. Map 7 (see Section 2.7) revealed a region in right occipital cortex showing a significant positive relationship between emotional response inhibition and CARE scores (see Table 5). Map 8 (see Section 2.7) showed a left occipital region exhibiting a significant positive relationship between emotional response inhibition and BIS scores (see Table 5). Measures of influence (Cook's distance) were below 1 for all participants for all of the above regions (see Supplementary Table 1). Correlation analyses revealed significant relationships between the emotional response inhibition contrast and several CARE subscales and BIS subscales in these regions (see Supplementary Table 5). Map 9 (conjunction of t-maps for emotional response inhibition contrast vs. CARE scores and vs. BIS scores) revealed no significant regions surviving correction for multiple comparisons.

**Table 5:** **Summary of significant regions from Maps 7 and 8**.

**Region**	***X***	***Y***	***Z***	**Volume**	**Regression vs. CARE**		**Regression vs. BIS**		***F*-test**	
	**(mm)**			**(mm^3^)**	***P***	***T***	***P***	***T***	***P***	***F***
**From Map 7: Emotional Response Inhibition Contrast vs CARE with CARE > BIS**									***F*-test CARE > BIS**	
Right Occipital Cortex	30.0	−82.0	13.0	5481.00	0.0239	2.29	0.1232	1.55	<0.0001	20.8138
**From Map 8: Emotional Response Inhibition Contrast vs BIS with BIS > CARE**									***F*-test BIS > CARE**	
Left Occipital Cortex	−21.0	−79.0	28.0	9639.00	0.2443	1.17	0.0198	2.37	<0.0001	21.6327

Summary of significant regions from Maps 7 and 8. Map 7 tested comparison of fMRI emotional response inhibition contrast (aversive NoGo−aversive Go) vs. CARE scores where CARE scores also accounted for more variance than BIS scores. Map 8 tested regression of emotional response inhibition contrast vs. BIS scores where BIS scores also accounted for more variance than CARE scores. X, Y, Z: MNI coordinates of region's peak statistical voxel. P- and t-values are median values across all voxels in each region (*df* = 98). F-test CARE > BIS values are median values across each region from the F-map testing whether CARE scores accounted for significantly more variance in emotional response inhibition contrast values than BIS scores (*df* = 1, 97). F-test BIS > CARE shows median values from the F-map testing whether BIS scores accounted for significantly more variance in emotional response inhibition contrast values than CARE scores (*df* = 1, 97). See Section 2.7 for details of analysis.

### 3.8. Outliers

As described at the end of Section 2.7, we excluded 23 regions from Maps 1, 2, 4, 5, 7, and 8 which did not retain significance after excluding outlier participants (two participants with smallest and largest CARE or BIS scores, or two participants with smallest or largest first-level fMRI contrast values). All retained regions exhibited maximum influence measures (maximum Cook's distance across all 19 participants) less than 1 (see Supplementary Table 1).

Three of the 19 participants were not right-handed (one left-handed, two ambidextrous). None of these three participants had the lowest or highest CARE or BIS scores. Their CARE scores were 2.82, 2.92, and 2.91, compared to the CARE score range of 1.66–4.06 for all 19 participants. Their BIS scores were 57, 68, and 50, compared to the BIS score range of 43–71. For the dorsomedial cerebellum region identified in Map 4 (see Table 4), an ambidextrous participant exhibited the highest value. For the dmPFC and pgACC regions identified in Map 5 (see Table 4), two non-right-handed participants exhibited the lowest emotional valence contrast values. Otherwise, the participants with the lowest and highest first-level contrast values were right-handed. Nonetheless, all regions presented above retained significance with outlier participants removed, as previously discussed. Based on visual inspection of first-level fMRI contrast values from the significant regions presented for Maps 1–9, the three non-right-handed participants did not display any consistent trend toward deviating from the other 16 right-handed participants in terms of fMRI contrast values. With only three non-right-handed participants, there was not enough statistical power to do a proper statistical comparison of right-handed vs. non-right-handed participants. We do not think that the inclusion of the three non-right-handed participants skewed the results presented here.

## 4. Discussion

Many studies have emphasized the role of impulsivity as a potential contributor to high-risk behavior tendencies (Levitt, 1991; Moore and Rosenthal, 1993; Luengo et al., 1994; Stanford et al., 1996; Ernst et al., 2006; Casey et al., 2008; Ernst and Mueller, 2008; Ernst and Fudge, 2009; Romer et al., 2009; Romer, 2010; Casey et al., 2010a; Dalley et al., 2011; Blakemore and Robbins, 2012). Accordingly, we expected to observe substantial similarities in how fMRI brain activation patterns from the emotional Go/NoGo task related to impulsivity and to risk behavior tendencies. Contrary to expectations, we found a dissociation between impulsivity and risk behavior tendencies in terms of fMRI activations. All but one of the regions detected in the statistical analyses reported in Sections 3.5 and 3.6 exhibited a significant relationship between fMRI first-level contrast amplitudes and either CARE risk scores or BIS impulsivity scores, but not both (see Tables 3, 4). *F*-tests comparing amounts of variance in fMRI contrast amplitude explained by CARE or BIS scores also supported this dissociation (see Tables 3, 4). The sole partial exception was one small cluster of voxels in right pOFC which showed an inverse relationship between emotional valence contrast and both CARE and BIS scores (see Table 4) although the relationship with CARE scores did not survive multiple comparison correction. Our results support the proposition that impulsivity and high-risk behavior tendencies are distinct (but related) constructs. Furthermore, a high impulsivity level is not equivalent to an elevated high-risk behavior tendency. We suggest that impulsivity may contribute to high-risk behavior in some cases but that greater impulsivity does not necessarily contribute to higher risk behavior tendencies.

High-risk behavior is complex, and it is acknowledged that various factors other than impulsivity are important potential contributors to risk tendency, such as reward seeking and sensation seeking (see Whiteside and Lynam, 2001; Romer et al., 2009; Romer, 2010; Dalley et al., 2011; Blakemore and Robbins, 2012). Relatively little emphasis has been placed on possible dissociations between high-risk behavior tendencies and impulsivity profiles. We are aware of one study that reported a dissociation between risk behavior tendencies as assessed using the Balloon Analog Risk Task and BIS impulsivity in a population of cigarette smokers (Ryan et al., 2013). Our observed dissociation is also consistent with studies that propose contributing factors to high-risk behavior other than impulsivity, such as reward seeking and sensation seeking (see Romer et al., 2009; Romer, 2010) as well as the influence of peers and social cues on behavior, particularly in adolescents (Gardner and Steinberg, 2005; Blakemore and Robbins, 2012).

The current study focused on impulsivity as measured by the BIS. Impulsivity is a complex construct; Bari and Robbins (2013) have suggested that impulsivity may involve multiple subdivisions of cognitive processes with as many as 9 distinct components (also see Whiteside and Lynam, 2001). Aspects of impulsivity not captured by the BIS Scale may show different relationships with fMRI activity patterns.

### 4.1. BIS impulsivity vs. aversive distractor valence ratings

Participants with higher BIS impulsivity scores rated aversive distractor images as being more unpleasant (lower valence scores, see Figure 3). This may reflect a greater sensitivity to aversive stimuli in more impulsive participants, commensurate with a possible contribution of negative urgency to higher impulsivity (see Whiteside and Lynam, 2001; Cyders and Smith, 2008).

### 4.2. Response inhibition activity vs. risk tendency and impulsivity

In right OFC and a region in vmPFC, participants with higher CARE risk scores exhibited lower response inhibition (NoGo−Go) contrast amplitude. These regions are not traditionally associated with motor response inhibition but rather with representing reward and value (see Mitchell, 2011). It is possible that, in participants with lower risk tendencies, successful completion of NoGo trials generated larger reward responses in these regions. Interestingly, our results in vmPFC and OFC are commensurate with findings in BPD. Diminished recruitment of BOLD activation during impulse control tasks has been reported in OFC and medial prefrontal regions in patients with BPD, a condition which is also associated with elevated high-risk behavior tendencies (Krause-Utz et al., 2014; Sebastian et al., 2014). We did find greater response inhibition-related activity for higher risk score individuals in right occipital cortex. It is possible that our emotional Go/NoGo task evoked greater attention responses in this region in those participants.

vlPFC has an established role in response inhibition (see Aron et al., 2004a, 2007; Dolcos et al., 2011; Mitchell, 2011). It has also been suggested that high-risk behavior tendencies might be caused by impulsivity as a result of reduced prefrontal behavioral control (see Ernst et al., 2006; Casey et al., 2008; Ernst and Mueller, 2008; Ernst and Fudge, 2009; Casey et al., 2010b). For these reasons, we expected to observe risk-related differences and/or impulsivity-related differences in response inhibition activation in vlPFC, but we did not observe this. In addition, Asahi et al. (2004) found that NoGo response inhibition-related activity was inversely correlated with individual impulsivity in a region they called right dlPFC. This region was actually located in the right inferior frontal sulcus on the border between right vlPFC and dlPFC. We expected to replicate this finding but did not. Horn et al. (2003) also previously found a relationship between reduced inhibition activation in the Go/NoGo task and increased participant impulsivity, in dmPFC. We did not replicate this finding, though we did find reduced emotional valence contrast amplitude in more impulsive participants in dmPFC as discussed below. Asahi et al. (2004) and Horn et al. (2003) both used a blocked design comparing blocks of pure Go trials with blocks of 50:50 mixed Go and NoGo trials. We used a rapid event-related design with an 80:20 ratio of Go:NoGo trials. These task design differences could account for differences between our results and those of Asahi et al. (2004) and Horn et al. (2003).

Conditions specific to poor impulse control in adolescents have also been examined in Go/NoGo fMRI studies (without a paired emotional task component). Risk behavior specific to impulsivity has been measured in drug naive adolescents diagnosed with ADHD (Rubia et al., 2005b), confirming a relationship between behavioral impulsiveness scores on screening questionnaires specific to the untreated disorder and reduced activation related to response inhibition in right vlPFC during the stop signal task. Adolescents at high-risk for developing Alcohol Use Disorder who later transitioned into heavy drinking adults also showed similarities between risk-based behavior and reduced brain activation related to response inhibition in various brain regions including vlPFC (Norman et al., 2011). Impulsive drinking behavior measured in heavy vs. light drinking college students has also indicated that those students who were prone to heavier episodes of drinking did show reduced fMRI activation during NoGo trials as compared to the students who did not drink as much alcohol in various brain regions including dlPFC (Ahmadi et al., 2013). In contrast, we did not observe any relationship between response inhibition-related activation in vlPFC or dlPFC and either CARE risk scores or Barratt impulsivity scores. Our participants exhibited low to medium CARE risk scores and low to medium Barratt impulsivity scores (see Section 3.1). It would be interesting to investigate whether including participants from the highest end of the risk and impulsivity spectra may reveal relationships between fMRI activation from the emotional Go/NoGo task and CARE or BIS scores in these regions.

### 4.3. Emotional processing and regulation vs. risk tendency and impulsivity

We observed reduced emotion-related activity (aversive−neutral distractor picture activation difference) in more impulsive participants in dmPFC, pgACC, and right pOFC. These regions have various roles in emotion-related processing and regulation as well as emotion-based response processing. Dorsomedial PFC (dmPFC) is involved in a variety of regulatory functions, recruited by various cognitive tasks. [Note: Some authors include dorsal ACC in dmPFC (e.g., Mitchell, 2011), whereas we take dmPFC to include only the medial aspect of the superior frontal gyrus excluding ACC.] dmPFC is thought to have roles in flexible behavior control, including resolution of response conflict and outcome value-related aspects of decision making (Venkatraman et al., 2009) and in emotion regulation (see Ochsner and Gross, 2005; Mitchell, 2011). Anterior cingulate cortex (ACC), inferiorly adjacent to dmPFC, has been implicated in performance monitoring, error detection, and process conflict monitoring (Carter et al., 1998; Botvinick et al., 2004; Botvinick, 2007; Mitchell, 2011). In particular, Carter et al. (1998) suggest that ACC may detect conditions that make errors more likely, such as the presence of emotional distractors in our emotional Go/NoGo task. dmPFC may also contribute to these functions (Stuss et al., 2001; Stemmer et al., 2004; Lvstad et al., 2012). pgACC is thought to be involved in processing salience related to emotion and motivation and is implicated in response preparation (Schulz et al., 2011). This region is also thought to provide regulatory control over emotion response circuitry in the amygdala and nucleus accumbens as well as autonomic functions in the hypothalamus and brainstem (Quirk and Gehlert, 2003; Ochsner and Gross, 2005; Mitchell, 2011, see). OFC is known to be involved in representing emotional stimulus value as well as learning and flexible control of emotions and of behavior in emotional contexts (Ochsner et al., 2002; O'Doherty, 2003; Ochsner et al., 2004; O'Doherty, 2004; Murray et al., 2007; O'Doherty, 2007; Wallis, 2007; Rolls and Grabenhorst, 2008; Dolcos et al., 2011; Mitchell, 2011; Golkar et al., 2012). Cyders et al. (2014b) found a relationship between increased fMRI activation for aversive stimulus processing in right lateral OFC, negative urgency (a component of impulsivity and subscale in the UPPS conception of impulsivity, see Whiteside and Lynam, 2001; Cyders and Smith, 2008), and general risk-taking. Negative urgency has also been associated with fMRI responses to alcohol-related cues in a ventromedial PFC region (Cyders et al., 2014a). Patients with BPD, which is associated with emotional dysregulation, show reduced fMRI activation in OFC, ACC, dmPFC, and dlPFC in response to emotion regulation tasks (Krause-Utz et al., 2014; Sebastian et al., 2014).

We interpret our findings in dmPFC, pgACC, and right pOFC as reflecting reduced recruitment of emotion regulation in these regions in more impulsive individuals. It is possible that reduced emotion regulation processing in all three regions could lead to increased impulsivity in a straight-forward manner. Participants with higher BIS impulsivity scores rated aversive distractor pictures as being more unpleasant (see Figure 3). Therefore, it seems unlikely that the reduced emotional valence contrast in dmPFC, pgACC, and right pOFC for higher BIS score participants could reflect a reduction in emotional stimulus salience in participants with higher impulsivity.

We observed greater aversive emotional valence contrast activation in right occipital cortex and dorsomedial cerebellum in participants with greater risk behavior tendencies. The meta-analysis by Kober et al. (2008) suggests that limbic projections enhance visual stream activity in the presence of emotional stimuli. Combined with changes in eye movement patterns, this results in changes to visual stream activity patterns. Kober et al. (2008) also point out that cerebellar efferents are more active during emotional states, possibly as the situational context portion of a larger pattern recognition network activated during emotional states. To our knowledge, ours is the first demonstration that emotion-related activity patterns in occipital cortex and dorsomedial cerebellum may be modulated by participant risk behavior tendency.

### 4.4. Emotional response inhibition activity vs. risk tendency and impulsivity

The emotional response inhibition contrast (aversive NoGo−aversive Go) did not show any significant relationships with CARE risk scores or BIS impulsivity scores in frontal regions. Several large clusters in vlPFC, dlPFC, and right anterior insula did show significantly larger activation for aversive NoGo vs. aversive Go trials, independently of CARE or BIS scores (see Supplementary Figure 4). Interestingly, Wilbertz et al. (2014) did not find differences related to high and low BIS scores in fMRI signals evoked by performance of the stop signal task, but they did find that participants' scores on the negative urgency subscale of the UPPS were negatively correlated with fMRI activation related to response inhibition in right vlPFC and anterior insula. Individuals scoring high for negative urgency are thought to exhibit impulsive decision-making specifically in the context of negative situations to escape from or minimize exposure to aversive stimuli (Whiteside and Lynam, 2001; Cyders and Smith, 2008). It would be informative to compare the fMRI emotional Go/NoGo task used here with UPPS subscores, including negative urgency, to further investigate this issue. It is also possible that inclusion of participants with more extreme risk and impulsivity tendencies may reveal relationships between emotional response inhibition fMRI activation and high risk or impulsivity tendencies. See discussion below in Section 4.6 for further details.

### 4.5. Negative BOLD responses

The vmPFC region from Map 1 (Figure 4) and pgACC region from Map 5 (Figure 5) exhibited negative BOLD signals (deactivation), with certain trial types inducing larger negative BOLD responses than others. The precise pattern of trial type-induced deactivation was modulated by CARE or BIS scores (see Sections 3.5, 3.6 for details). In vmPFC from Map 1, participants with higher CARE risk scores exhibited greater negative BOLD deflection for NoGo trials, while lower CARE score participants showed the opposite pattern. In pgACC from Map 5, higher BIS score participants exhibited greater negative BOLD deflection for aversive compared to neutral distractor trials, while low BIS participants showed the converse pattern. The vmPFC and pgACC regions are part of the default mode or task negative network, which is well known to show negative BOLD responses when participants perform focused tasks such as the emotional Go/NoGo task (see Raichle et al., 2001; Buckner et al., 2008; Andrews-Hanna et al., 2010; Buckner, 2012). It has been suggested that medial prefrontal (and posterior cingulate) parts of the default mode network are involved in self-referential, emotional decisions and regulation (Andrews-Hanna et al., 2010). It is possible that the fMRI activation patterns described above reflect a deeper disengagement of self-referential functions in the anterior medial default mode network in higher CARE score participants engaged in response inhibition in NoGo trials and in higher BIS score participants performing trials with aversive distractors.

### 4.6. Limitations and future directions

Our task included aversive but not pleasant emotional stimuli. Both Casey et al. (2010b) and Ernst and Fudge (2009) have suggested that higher risk behavior tendencies are associated with increased responsivity to pleasant, rewarding stimuli. Many risk behaviors, such as high-risk sex, recreational drug use, and excess alcohol consumption, are pursued in part for their rewarding properties, and the current study design would not assess reward-related aspects of risk-related neuronal processing. A future fMRI study using an emotional Go/NoGo task with positive, aversive, and neutral distractors would allow investigation of reward- and approach- as well as avoidance-related brain activity patterns as they relate to risk behavior tendencies and impulsivity. In addition, participants in the current study fell into low- to medium-risk and low- to medium-impulsivity categories. A future fMRI study recruiting participants with a broader range of risk behavior tendencies and impulsivity levels would allow a more complete picture of brain activity patterns related to risk and impulsivity.

We focused on impulsivity measures provided by the BIS. In the introduction, we discussed the complexity of the impulsivity construct and the lack of a single consensus on how to define that construct. It would be informative to extend the approach used here to include alternative measures of impulsivity as well as related concepts such as sensation and reward seeking from other psychometric instruments including the SSS (Zuckerman, 1994), UPPS (Whiteside and Lynam, 2001), TPQ (Cloninger et al., 1991), and the I7 (Eysenck et al., 1985).

We assessed risk-behavior tendencies using the CARE questionnaire, which provides the advantage that it addresses a wide range of real-world risk behaviors that are influenced by emotions (unsafe sex, alcohol binging, and so on). That the CARE is a self-report instrument raises the possibility that participants may distort their answers, for example by under-reporting socially-undesirable risk behaviors. We note, however, that risk behavior scores from the CARE questionnaire have been shown to associate strongly with objective risk-related measures such as frequency of emergency room visits due to alcohol misuse (Kelly et al., 2005). It would also be informative to combine the approach used here with objective risk behavior measures such as those from the balloon analog risk task (BART; Lejuez et al., 2002) or risky decision-making tasks or gambling tasks from the neuroeconomics literature (for review, see Loewenstein et al., 2008). Though these tasks do not directly assess the same real-world risk behaviors that are the focus of this article, these tasks do provide objective measures of risk-related decision-making.

### 4.7. Conclusions

We investigated differences related to participants' risk behavior tendencies and impulsivity levels in fMRI brain activity patterns evoked by an emotional Go/NoGo task. We focused on impulsivity as it is assessed using the BIS instrument and risk behavior tendencies measured using the CARE self-report instrument. Our results showed a dissociation between brain activity profiles related to CARE risk tendencies and to BIS impulsivity, supporting a view of BIS impulsivity and high-risk behavior tendencies as distinct constructs. This view is consistent with previous suggestions that risk behavior can be driven not just by impulsivity as assessed using the BIS, which emphasizes cognitive processes, executive control, and response inhibition, but also by other factors such as reward seeking or sensation seeking (Romer, 2010) and by social behaviors (Gardner and Steinberg, 2005; Blakemore and Robbins, 2012). Higher BIS impulsivity levels may contribute to increased risk behavior tendencies in some cases, but elevated BIS impulsivity is not equivalent to elevated risk behavior tendency. Our results suggest that treatment for high-risk behavior in highly impulsive patients may be more effective if a nuanced approach is taken to understanding potential multi-faceted causes of the high-risk behavior, rather than attributing it to high impulsivity from poor cognitive control.

### Conflict of interest statement

The authors declare that the research was conducted in the absence of any commercial or financial relationships that could be construed as a potential conflict of interest.

## Footnotes

^1^Note on anatomical terminology: To make the manuscript more accessible to readers outside the brain imaging/neuroanatomy subfields, we employ widely-used, broad subdivisions of prefrontal cortex including vlPFC, dlPFC, vmPFC, and dmPFC, as opposed to anatomically more specific terms such as inferior, middle, and superior frontal gyri. It has been pointed out, however, that precise definitions of these broad prefrontal subdivisions exhibit some variability in terms of extent and anatomical boundaries across different studies (for example, see Cieslik et al., 2013).
