# Supplementary material for: Neural correlates of high-risk behavior tendencies and impulsivity in an emotional Go/NoGo fMRI task
Source: Front Syst Neurosci. 2015 Mar 10;9:24. doi: 10.3389/fnsys.2015.00024 (PMC4354310; doi:10.3389/fnsys.2015.00024)
Supplement: Supplementary file 1 [file Presentation1.PDF]

Supplementary Material: Neural correlates of high-risk behaviour tendencies and impulsivity in an emotional Go/NoGo fMRI task

Matthew R. G. Brown [1,*], James R. A. Benoit [1], Michal Juhas [1], R. Marc Lebel [3,4], Marnie MacKay [1], Ericson Dametto [1], Peter H. Silverstone [1], Florin Dolcos [1,2], Serdar M. Dursun [1], Andrew J. Greenshaw [1]

[1] *Dept. of Psychiatry, University of Alberta, Edmonton, Alberta, Canada*
[2] *Dept. of Psychology, Neuroscience Program, and the Beckman Institute for Advanced Science and Technology, University of Illinois Urbana-Champaign, Illinois, USA*
[3] *Dept. of Biomedial Engineering, University of Alberta, Edmonton, Alberta, Canada*
[4] *Now at General Electric*

Correspondence*:
Matthew R. G. Brown
University of Alberta, Department of Psychiatry, 12-127A Clinical Sciences Building, Edmonton, Alberta, T6G2B3, Canada, mbrown2@ualberta.ca

1 SUPPLEMENTARY METHODS

1.1 DETAILS OF SECOND-LEVEL STATISTICAL COMPARISONS

List of second-level statistical maps:

- **Map 1:** response inhibition contrast vs. CARE scores with CARE > BIS,
- **Map 2:** response inhibition contrast vs. BIS scores with BIS > CARE,
- **Map 3:** response inhibition contrast vs. CARE AND BIS scores,
- **Map 4:** emotional valence contrast vs. CARE scores with CARE > BIS,
- **Map 5:** emotional valence contrast vs. BIS scores with BIS > CARE,
- **Map 6:** emotional valence contrast vs. CARE AND BIS scores.
- **Map 7:** emotional response inhibition contrast vs. CARE scores with CARE > BIS,
- **Map 8:** emotional response inhibition contrast vs. BIS scores with BIS > CARE,
- **Map 9:** emotional response inhibition contrast vs. CARE AND BIS scores.

The nine second-level comparisons described above were computed as follows. For each of the three first level contrasts (response inhibition contrast, emotional valence contrast, emotional response inhibition contrast), two second-level GLMs were fit using the mixed-effects method of Worsley et al. (2002). These GLMs modeled the linear relationship between either CARE risk scores or BIS impulsivity scores and the first-level contrast magnitude. That is, the second-level design matrix included one constant offset

predictor column and one column with either the mean-centred CARE scores or the mean-centred BIS scores for all 19 participants. T-tests on the fitted weight value for the CARE or BIS score column allowed the construction of statistical t-maps.

Maps 1, 4, and 7 were computed as follows. For a given first level contrast, a statistical t-map was computed testing first level contrast values vs. CARE scores using the appropriate GLM. The t-map was thresholded voxelwise at $p < 0.05$ ($|t| > 1.984$, two-tailed, df=98). A cluster mass threshold of 465 was applied to the t-map to achieve global correction for multiple comparisons at $p < 0.05$ across the voxel population and across all six second-level statistical comparisons. Then the t-map was conjoined with an F-test testing CARE > BIS. Thus, only voxels showing a significant relationship with CARE scores, where CARE scores also accounted for significantly more variance than BIS scores, were retained. We refer to the F-test testing whether CARE scores accounted for significantly more variance in first level contrast values than did BIS scores as F-test CARE > BIS. For this F-test, the restricted GLM model consisted of a constant offset column and a column of mean-centred BIS scores (i.e. identical to the GLM for regression against BIS scores described above). The unrestricted GLM model consisted of the columns in the restricted model as well as a column of mean-centred CARE scores. Residuals from the restricted and unrestricted models were compared to derive an F-statistic (at each voxel location) in the standard way (see Draper and Smith, 1998). The resulting F-map was thresholded voxelwise at $p < 0.05$ ($F > 3.939$, df=1, 97). As described above, the thresholded F-map was then conjoined with the t-map from the GLM regression of CARE scores against the first-level contrast of interest.

Maps 2, 5, and 8 were computed as described in the previous paragraph but with the roles of CARE and BIS scores swapped in the obvious manner.

For Maps 3, 6, and 9, statistical t-maps from regressions of first level contrast values against CARE and against BIS scores were thresholded voxelwise at $p < 0.05$ ($|t| > 1.984$, two-tailed, df=98). The t-maps were conjoined such that only voxels showing significance in both maps were retained. To correct for multiple comparisons at $p < 0.05$, a cluster mass threshold of 465 was applied to the conjunction map, using t-values from the contrast vs. CARE map to compute cluster mass. (Using t-values from the contrast vs. BIS map to compute cluster mass did not change the results.) No voxels survived multiple comparison correction in Map 3, 6, or 9.

1.2 ANALYSIS OF CARE AND BIS SUBSCALES

We performed an analysis of relative contributions of the six CARE subscales or six BIS subscales to the fMRI contrast vs. CARE score and fMRI contrast vs. BIS score results. We performed region of interest (ROI) analyses for each of the 12 significant clusters that survived statistical testing and quality assurance filtering (see fMRI Analysis section in main text). For each region, first-level GLMs were fit to the mean activation timecourse for each participant. Then second-level GLMS were fit using one of the CARE or BIS subscales as the regressor in the second-level design matrix. This was done for each of the six CARE subscales or six BIS subscales, depending on whether the region being analysed was derived from one of the fMRI contrast vs. CARE score analyses or fMRI contrast vs. BIS score analyses. These analyses are not independent of the whole brain analyses that selected the regions in the first place. Potential dangers from double-dipping have been pointed out previously (Vul et al., 2009; Kriegeskorte et al., 2009). These analyses are included for exploratory purposes.

1.3 PARTICIPANTS' STATISTICAL INFLUENCE ON MAPS 1-9

For each region identified in Maps 1-9, we computed the statistical influence (Cook's distance D; Cook and Weisberg, 1982) that each participant's data point exerted on the second-level linear regression analysis of CARE or BIS scores against the relevant first-level fMRI contrast values. Cook and Weisberg (1982) suggest that a D value > 1 indicates a data point with high statistical influence. Results are presented in Supplementary Table 1.

2 SUPPLEMENTARY TABLES AND FIGURES

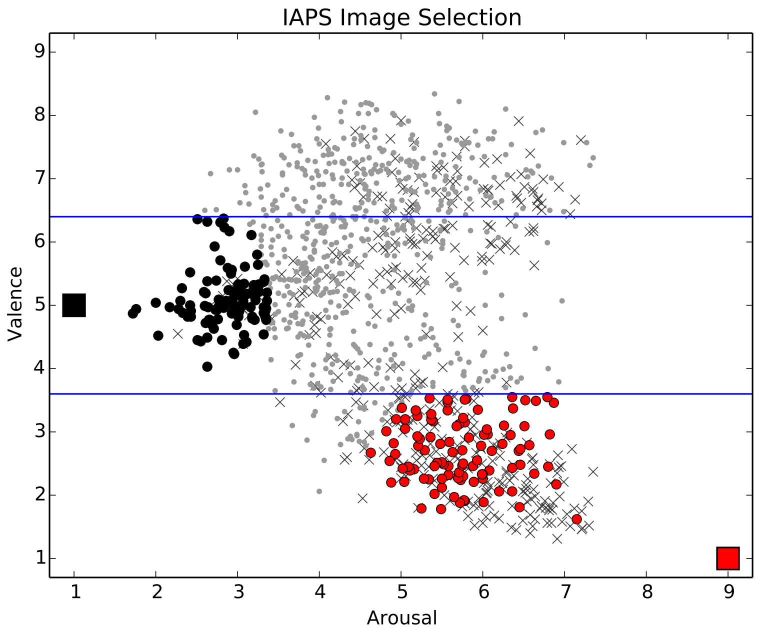

Supplementary Figure 1. Scatterplot of arousal and valence scores for all images in the IAPS picture set. Black and red dots are the images selected as neutral and aversive distractors, respectively. The large black and red squares are the targets for neutral and aversive distractors, respectively (see main text section 2.3). Small gray dots are unused IAPS images. Gray x's are images rejected by the two psychiatrists (see main text section 2.3). Blue horizontal lines mark valence cutoff values of 3.6 and 6.4 for selecting aversive and neutral distractors.

Response Inhibition Contrast (independent of CARE and BIS scores)

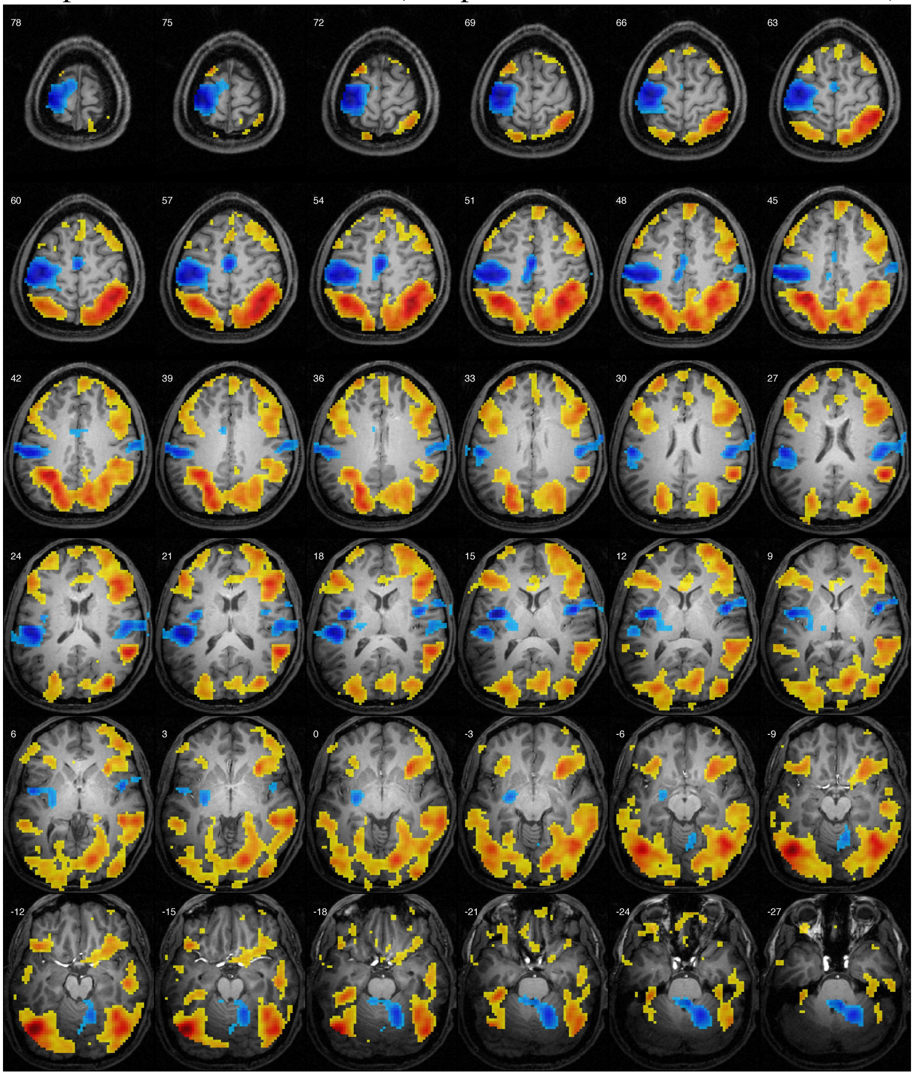

Supplementary Figure 2. Statistical map for response inhibition contrast (NoGo - Go, collapsed across distractor valence), independent of CARE or BIS scores. $p<0.05$, corrected for multiple comparisons across voxel population. Neurological convention (left of image is left of brain). Axial slices' MNI coordinates in mm shown in upper-left.

Emotional Valence Contrast (independent of CARE and BIS scores)

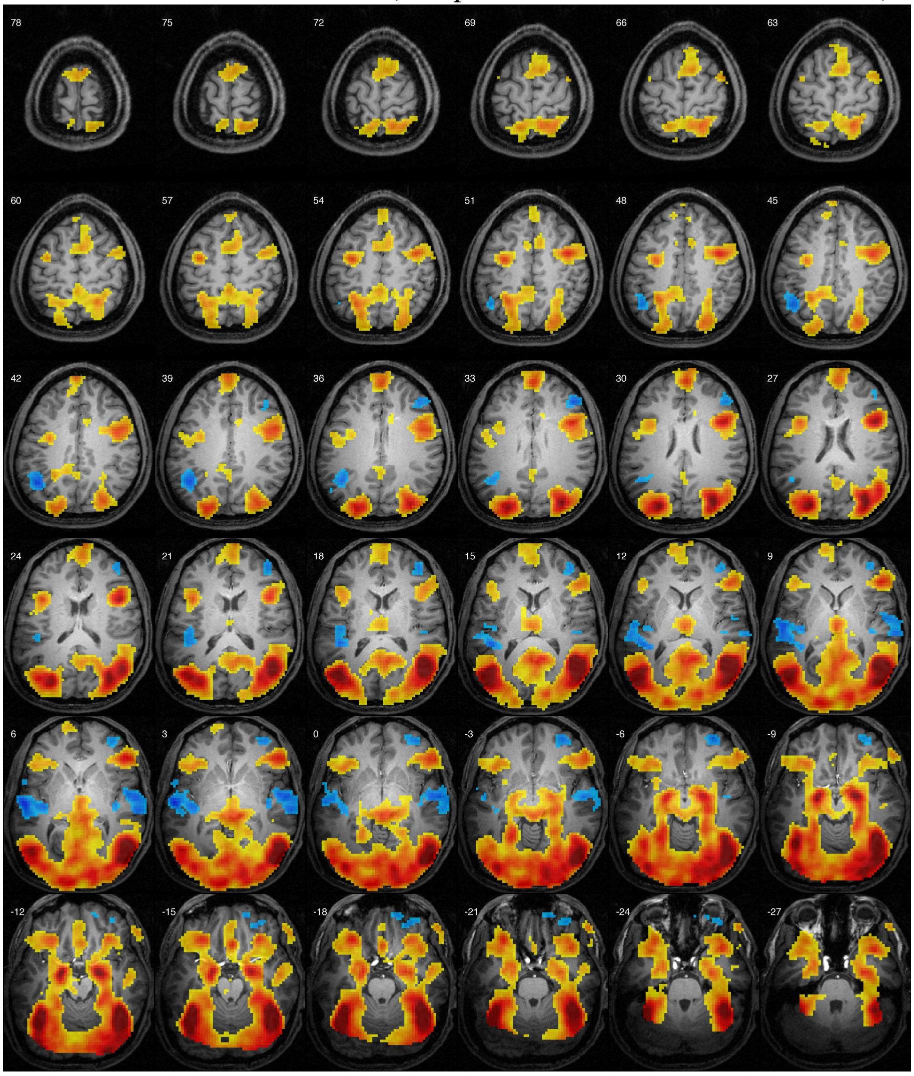

Supplementary Figure 3. Statistical map for emotional valence contrast (aversive - neutral distractor trials, collapsed across NoGo vs. Go), independent of CARE or BIS scores. $p<0.05$, corrected for multiple comparisons across voxel population. Neurological convention (left of image is left of brain). Axial slices' MNI coordinates in mm shown in upper-left.

Emotional Response Inhibition Contrast (independent of CARE and BIS scores)

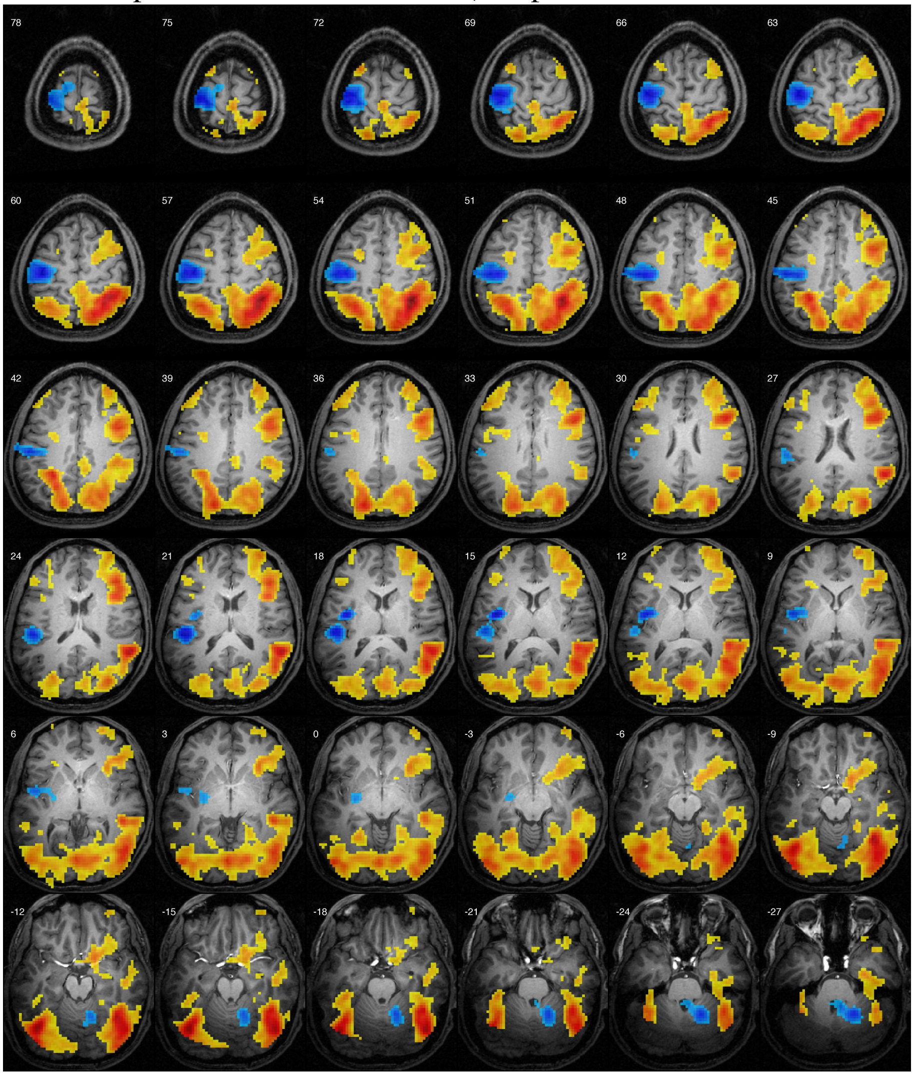

Supplementary Figure 4. Statistical map for emotional response inhibition contrast (aversive NoGo - aversive Go), independent of CARE or BIS scores. $p<0.05$, corrected for multiple comparisons across voxel population. Neurological convention (left of image is left of brain). Axial slices' MNI coordinates in mm shown in upper-left.

Supplementary Table 1. Influence measures for Maps 1-9

ROI Name	D_Max	D_01	D_02	D_03	D_04	D_05	D_06	D_07	D_08	D_09	D_10	D_11	D_12	D_13	D_14	D_15	D_16	D_17	D_18	D_19
**From Map 1** (response inhibition vs. CARE)																				
Right OFC	0.295	0.010	0.002	0.154	0.050	0.004	0.295	0.001	0.001	0.016	0.137	0.012	0.063	0.084	0.214	0.007	0.005	0.068	0.121	0.007
vmPFC	0.760	0.040	0.063	0.000	0.020	0.002	0.124	0.001	0.049	0.008	0.038	0.760	0.018	0.001	0.205	0.004	0.030	0.173	0.012	0.012
Right Occipital	0.247	0.020	0.110	0.101	0.064	0.036	0.003	0.003	0.000	0.026	0.175	0.140	0.053	0.014	0.247	0.003	0.060	0.003	0.173	0.070
**From Map 4** (emotional valence vs. CARE)																				
Right Occipital	0.422	0.012	0.000	0.027	0.014	0.075	0.028	0.001	0.048	0.032	0.004	0.040	0.025	0.002	0.422	0.114	0.014	0.155	0.007	0.000
dmCereb	0.276	0.002	0.093	0.022	0.055	0.007	0.016	0.018	0.054	0.047	0.013	0.276	0.093	0.039	0.060	0.119	0.000	0.013	0.031	0.033
**From Map 5** (emotional valence vs. BIS)																				
dmPFC	0.410	0.001	0.018	0.332	0.000	0.037	0.001	0.016	0.328	0.043	0.070	0.003	0.002	0.045	0.048	0.071	0.053	0.000	0.410	0.005
pgACC	0.172	0.001	0.172	0.102	0.027	0.061	0.009	0.002	0.126	0.022	0.013	0.003	0.040	0.004	0.004	0.169	0.017	0.021	0.000	0.073
Right pOFC	0.726	0.000	0.031	0.276	0.726	0.146	0.012	0.008	0.001	0.003	0.069	0.203	0.005	0.081	0.035	0.050	0.012	0.011	0.014	0.003
Right Temp Pole	0.723	0.016	0.008	0.010	0.040	0.723	0.001	0.215	0.002	0.085	0.046	0.030	0.004	0.002	0.002	0.019	0.026	0.065	0.107	0.051
**From Map 7** (emotional response inhibition vs. CARE)																				
Right Occipital	0.337	0.042	0.036	0.052	0.000	0.001	0.227	0.062	0.000	0.001	0.062	0.205	0.031	0.066	0.337	0.003	0.079	0.013	0.141	0.015
**From Map 8** (emotional response inhibition vs. BIS)																				
Left Occipital Cortex	0.551	0.015	0.013	0.551	0.549	0.024	0.002	0.015	0.007	0.027	0.120	0.014	0.003	0.018	0.000	0.021	0.116	0.090	0.182	0.007

Measures of influence (Cook's distance D) computed for linear regression of either CARE or BIS scores against participants' fMRI first-level contrast values, for significant regions from Maps 1-9. D_Max is maximum D across all participants. D_i is the D value for participant i. Cook and Weisberg (1982) suggest that a D value ≤ 1 represents low statistical influence. See Supplementary Methods section 1.3 for methodological details.

Supplementary Table 2. CARE subscale analysis for significant regions from Map 1

Region	Correlations for Response Inhibition Contrast with CARE Subscales						
	Overall	Drug	Illegal	Sexual	Drinking	Sports	Work
**From Response Inhibition Contrast vs CARE Score Analysis**							
Right OFC	-0.69**	-0.14	-0.40†	0.04	-0.41†	-0.51*	-0.52*
vmPFC	-0.66**	-0.22	-0.54*	-0.21	-0.49*	-0.10	-0.48*
Right Occipital	0.61**	0.26	0.49*	-0.06	0.55*	0.04	0.57*

CARE subscale analysis for regions identified in Map 1 and listed in Table 3 in the main text. These regions were identified from comparison of fMRI response inhibition contrast (NoGo - Go) vs. CARE overall risk score. Values shown are correlations between response inhibition contrast and CARE overall score and CARE subscale scores. Positive and negative correlation values, respectively, indicate positive and negative relationships between fMRI contrast amplitude and the relevant subscale score. Overall: mean of all six CARE subscales. Drug: Illicit Drug Use subscale. Illegal: Aggressive / Illegal Behaviours subscale. Sexual: Risky Sexual Activities subscale. Drinking: Heavy Drinking subscale. Sports: High Risk Sports subscale. Work: Academic/Work Behaviours subscale. See Fromme et al. (1997) for details of CARE questionnaire. OFC: orbitofrontal cortex. vmPFC: bilateral ventromedial prefrontal cortex. Occipital: occipital cortex. † $p<0.1$, * $p<0.05$, ** $p<0.01$, *** $p<0.001$ (df = 17, two-tailed, uncorrected).

Supplementary Table 3. CARE subscale analysis for significant regions from Map 4

Region	Correlations with CARE Subscales						
	Overall	Drug	Illegal	Sexual	Drinking	Sports	Work
**From Emotional Valence Contrast vs CARE Score Analysis**							
Right Occipital	0.78***	0.24	0.57*	-0.02	0.57*	0.37	0.55*
dmCereb	0.61**	0.17	0.30	0.50*	0.63**	-0.01	0.08

CARE subscale analysis for regions from Map 4, listed in upper part of Table 4 in the main text. These regions were identified from comparison of fMRI emotional valence contrast (aversive - neutral distractor images) vs. CARE overall risk score. Values shown are correlations between emotional valence contrast and CARE overall score and CARE subscale scores. Positive and negative correlation values, respectively, indicate positive and negative relationships between fMRI contrast amplitude and the relevant subscale score. Overall: mean of all six CARE subscales. Drug: Illicit Drug Use subscale. Illegal: Aggressive / Illegal Behaviours subscale. Sexual: Risky Sexual Activities subscale. Drinking: Heavy Drinking subscale. Sports: High Risk Sports subscale. Work: Academic/Work Behaviours subscale. See Fromme et al. (1997) for details of CARE questionnaire. Occipital: occipital cortex. dmCereb: dorsomedial cerebellum. † $p<0.1$, * $p<0.05$, ** $p<0.01$, *** $p<0.001$ (df = 17, two-tailed, uncorrected).

Supplementary Table 4. BIS subscale analysis for significant regions from Map 5

Region	Correlations with BIS Subscales						
	Overall	Attention	Cog Instability	Motor	Perseverance	Self-control	Cog Complexity
**From Emotional Valence Contrast vs BIS Score Analysis**							
dmPFC	-0.69**	-0.62**	-0.52*	-0.46*	-0.39†	-0.53*	-0.03
pgACC	-0.62**	-0.81***	-0.53*	-0.38	-0.12	-0.45†	0.13
Right pOFC	-0.74***	-0.62**	-0.37	-0.32	-0.28	-0.50*	-0.56*
Right Temp Pole	-0.72***	-0.46*	-0.52*	-0.34	-0.33	-0.62**	-0.36

BIS subscale analysis for regions from Map 5, listed in lower part of Table 4 in the main text. These regions were identified from comparison of fMRI emotional valence contrast (aversive - neutral distractor images) vs. overall BIS risk score. Values shown are correlations between emotional valence contrast and BIS overall score and BIS subscale scores. Positive and negative correlation values, respectively, indicate positive and negative relationships between fMRI contrast amplitude and the relevant subscale score. Overall: overall BIS score (sum of subscales). Attention: Attentional 1st order subscale. Cog Instability: Cognitive Instability 1st order subscale. Motor: Motor 1st order subscale. Perseverance: Perseverance 1st order subscale. Self-control: Self-control 1st order subscale. Cog Complexity: Cognitive Complexity 1st order subscale. See Patton et al. (1995) for details of BIS questionnaire. dmPFC: dorsomedial prefrontal cortex. pgACC: perigenual anterior cingulate cortex. pOFC: posterior orbitofrontal cortex. Temp Pole: temporal pole. † $p<0.1$, * $p<0.05$, ** $p<0.01$, *** $p<0.001$ (df = 17, two-tailed, uncorrected).

Supplementary Table 5. CARE and BIS subscale analyses for significant regions from Maps 7 and 8

Region	Map 7 Regions' Correlations with CARE Subscales					
	Overall CARE	Drug	Illegal	Sexual	Drinking	Sports	Work
**From Emotional Response Inhibition Contrast vs CARE Score Analysis**							
Right Occipital	0.74***	0.53*	0.35	-0.11	0.10	-0.32	0.38

Region	Map 8 Regions' Correlations with BIS Subscales						
	Overall BIS	Attention	Cog Instability	Motor	Perseverance	Self-control	Cog Complexity
**From Emotional Response Inhibition Contrast vs BIS Score Analysis**							
Left Occipital	0.75***	0.00	-0.27	-0.08	-0.62**	-0.36	0.23

CARE and BIS subscale analyses for regions from Maps 7 and 8, respectively, listed in Table 5 in the main text. These regions were identified from comparison of fMRI emotional response inhibition contrast (aversive NoGo - aversive Go) vs. overall CARE risk and BIS impulsivity scores. Top: Values shown are correlations between emotional response inhibition contrast and CARE overall score or subscale scores. Bottom: Values shown are correlations between emotional response inhibition contrast and BIS overall score or subscale scores. Positive and negative correlation values, respectively, indicate positive and negative relationships between fMRI contrast amplitude and the relevant subscale score. Overall CARE: mean of all six CARE subscales. Drug: Illicit Drug Use CARE subscale. Illegal: Aggressive / Illegal Behaviours CARE subscale. Sexual: Risky Sexual Activities CARE subscale. Drinking: Heavy Drinking CARE subscale. Sports: High Risk Sports CARE subscale. Work: Academic/Work Behaviours CARE subscale. See Fromme et al. (1997) for details of CARE questionnaire. Overall BIS: overall BIS score (sum of subscales). Attention: Attentional 1st order BIS subscale. Cog Instability: Cognitive Instability 1st order BIS subscale. Motor: Motor 1st order BIS subscale. Perseverance: Perseverance 1st order BIS subscale. Self-control: Self-control 1st order BIS subscale. Cog Complexity: Cognitive Complexity 1st order BIS subscale. See Patton et al. (1995) for details of BIS questionnaire. dmPFC: dorsomedial prefrontal cortex. pgACC: perigenual anterior cingulate cortex. pOFC: posterior orbitofrontal cortex. Temp Pole: temporal pole. † $p<0.1$, * $p<0.05$, ** $p<0.01$, *** $p<0.001$ (df = 17, two-tailed, uncorrected).

REFERENCES

Cook, R. D., Weisberg, S., 1982. Residuals and Influence in Regression. Chapman and Hall.

Draper, N. R., Smith, H., 1998. Applied Regression Analysis. John Wiley.

Fromme, K., Katz, E. C., Rivet, K., Aug. 1997. Outcome expectancies and risk-taking behavior. Cognitive Therapy And Research 21 (4), 421–442.

Kriegeskorte, N., Simmons, W. K., Bellgowan, P. S. F., Baker, C. I., May 2009. Circular analysis in systems neuroscience: the dangers of double dipping. Nat Neurosci 12 (5), 535–540.

Patton, J. H., Stanford, M. S., Barratt, E. S., Nov 1995. Factor structure of the Barratt Impulsiveness Scale. J Clin Psychol 51 (6), 768–774.

Vul, E., Harris, C., Winkielman, P., Pashler, H., 2009. Puzzlingly high correlations in fMRI studies of emotion, personalit1y, and social cognition. Perspectives on Psychological Science 4 (3), 274–290.

Worsley, K. J., Liao, C. H., Aston, J., Petre, V., Duncan, G. H., Morales, F., Evans, A. C., Jan 2002. A general statistical analysis for fMRI data. Neuroimage 15 (1), 1–15.
